# A Systematic Review and Meta-Analysis of Minimally Invasive Partial Nephrectomy Versus Focal Therapy for Small Renal Masses

**DOI:** 10.3389/fonc.2022.732714

**Published:** 2022-05-26

**Authors:** Lin Dong, Wang You Liang, Lu Ya, Liu Yang, Wei Qiang

**Affiliations:** ^1^ Department of Urology, Pengzhou People’s Hospital, Chengdu, China; ^2^ Department of Laboratory, Pengzhou People’s Hospital, Chengdu, China; ^3^ Department of Respiratory and Critical Care Medicine, First Affiliated Hospital of Chengdu Medical College, Chengdu, China; ^4^ Department of Urology, West China Fourth Hospital of Sichuan University, Chengdu, China

**Keywords:** minimally invasive, focal therapy, ablation techniques, kidney, nephrectomy, meta-analysis

## Abstract

**Background:**

Minimally invasive partial nephrectomy (MIPN) and focal therapy (FT) are popular trends for small renal masses (SRMs). However, there is currently no systematic comparison between MIPN and FT of SRMs. Therefore, we systematically study the perioperative, renal functional, and oncologic outcomes of MIPN and FT in SRMs.

**Methods:**

We have searched the Embase, Cochrane Library, and PubMed for articles between MIPN (robot-assisted partial nephrectomy and laparoscopic partial nephrectomy) and FT {radiofrequency ablation (RFA), microwave ablation (MWA), cryoablation (CA), irreversible electroporation, non-thermal [irreversible electroporation (IRE)] ablation, and stereotactic body radiation therapy (SBRT)}. We calculated pooled mean difference (MD), odds ratios (ORs), and 95% confidence intervals (CIs) (CRD42021260787).

**Results:**

A total of 26 articles (n = 4,420) were included in the study. Compared with MIPN, the operating time (OP) of FT had significantly lower (SMD, −1.20; CI, −1.77 to −0.63; I^2^ = 97.6%, P < 0.0001), estimated blood loss (EBL) of FT had significantly less (SMD, −1.20; CI, −1.77 to −0.63; I^2^ = 97.6%, P < 0.0001), length of stay (LOS) had shorter (SMD, −0.90; CI, −1.26 to −0.53; I^2^ = 92.2%, P < 0.0001), and estimated glomerular filtration rate (eGFR) of FT was significantly lower decrease (SMD, −0.90; CI, −1.26 to −0.53; I^2^ = 92.2%, P < 0.0001). However, FT possessed lower risk in minor complications (Clavien 1–2) (OR, 0.69; CI, 0.45 to 1.07; I^2^ = 47%, P = 0.023) and overall complications (OR, 0.71; CI, 0.51 to 0.99; I^2^ = 49.2%, P = 0.008). Finally, there are no obvious difference between FT and MIPN in local recurrence, distant metastasis, and major complications (P > 0.05).

**Conclusion:**

FT has more advantages in protecting kidney function, reducing bleeding, shortening operating time, and shortening the length of stay. There is no difference in local recurrence, distant metastasis, and major complications. For the minimally invasive era, we need to weigh the advantages and disadvantages of all aspects to make comprehensive choices.

**Systematic Review Registration:**

https://www.crd.york.ac.uk/PROSPERO/#recordDetails, identifier PROSPERO (CRD42021260787).

## Introduction

Small renal masses (SRMs) represent a group of heterogeneous tumors covering the entire metastatic potential, including malignant, indolent, and benign tumors. Among them, kidney cancer already accounts for 2%–3% of all cancers, and the incidence is increasing year by year (1, 2). The American Urological Association (AUA) and European Association of Urology (EAU) guidelines both recommend partial nephrectomy for SRMs (3). In addition, minimally invasive partial nephrectomy (MIPN) including robot-assisted partial nephrectomy (RAPN) and laparoscopic partial nephrectomy (LPN) is the current trend. However, OPN is selected, most of which are intraoperatively converted from LPN or RAPN to OPN for SRMs. In addition, it has recently been fully developed in the clinic.

With the clinical application of ablation techniques, focal therapy (FT) {radiofrequency ablation (RFA), microwave ablation (MWA), cryoablation (CA), irreversible electroporation, and non-thermal [irreversible electroporation (IRE)] ablation} has been fully developed (4). FT has the advantages of less trauma, less bleeding, and shorter hospital stay (5). The guidelines of AUA and EUA recommend FT replacing PN for kidney mass < 3 cm in size, and it is suitable for patients with kidney masses who are forbidden to operate or have serious comorbidities (6, 7). Therefore, the comparative study on MIPN and FT is very meaningful. However, there are main systematic reviews about ablative therapies versus partial nephrectomy for small renal masses at present (8). Therefore, the purpose of this study is to compare the perioperative period, renal function, and oncologic outcomes of MIPN and FT in SRMs.

## Methods

### Protocol and Guidance

The study was performed according to Preferred Reporting Items for Systematic Reviews and the meta-analysis (PRISMA) (9). The protocol for this review has been registered on PROSPERO (CRD42021260787).

### Search Strategy

This study involved literature published in the Embase, PubMed, and Cochrane Library up to January 26, 2022. We defined the eligibility criteria according to the population (P), intervention (I), comparator (C), outcome, and study design approach (O). P, the patients with SRMs; I, undergoing MIPN; C, FT was performed as a comparator; O, one or more of the following outcomes: perioperative, renal functional, and oncologic outcomes. The search terms included (robot-assisted partial nephrectomy OR robotic partial nephrectomy OR RAPN OR RPN OR laparoscopic partial nephrectomy OR LPN OR Minimally invasive) AND [“Renal Neoplasm” (MeSH) OR renal masses] AND [“radiofrequency ablation” (MeSH) OR “Cryoablation” (MeSH) OR microwave ablation OR RFA OR irreversible electroporation OR CRA OR MWA OR IEP OR “Stereotactic body radiation therapy” (MeSH) OR SBRT]. The search strategy was not limited by language or year. It was not requested by the ethics or institutional review committee due to the study being designed as a systematic review and meta-analysis.

### Inclusion and Exclusion Criteria

We have included the literature by the following criteria. Comparative data were available on the treatment of SRMs through MIPN (RAPN and LPN) and FT (RFA, CA, MWA, and IRE). Outcome indexes should include at least one of the following: perioperative period, renal function, and oncologic outcomes. Any study that did not confirm the above inclusion criteria was excluded.

### Data Extraction and Outcome Measures

Two researchers (LY and LX) independently have reviewed the retrieved literature by the inclusion and exclusion criteria. The third researcher (ZZJ) was asked to participate in the discussion to decide whether to include when disagreements were encountered. The extracted data included the first author, publication, country, study type, group, age (if mentioned), follow-up, female proportion, and renal nephrectomy score (**Table 1**).

**Table 1:** Characteristics of the included studies.

Author	Year	End points	Publication	Country	Study design	Study interval	Group	Cases	Malignant tumour, n (%)	Tumour grade (1-2),n (%)	Clinical T1, n(%)	Clavien grade (0-2), n (%)	Age	Male proportion(%)	BMI(Body mass index) (kg/m2)	Comorbidities ASA(%)	tumour size(cm)	Pathology (Ma/Be/Un)	R.E.N.A.L Nephrometry score	Follow-up (months)	Confounders adjustment	NOS score(max:9)
Bensalah (10)	2007	Survival, recurrence, complications	BJU international	USA	R	2000-2006	LPN	50	41 (82)	37 (90)			56.5 ± 11.7	62	31.1 ± 8.0	≥3(53%)	2.6 ± 0.9	N/A	N/A	25	No	8
							LRFA	38	29 (80)	20 (95)			62 ± 17.5	58	29.6 ± 4.8	≥ 3(26%)	2.3 ± 0.7			15		
Bertolo (11)	2019	Survival, recurrence, complications, renal function	Urologic Oncology	USA	R	2006-2016	RAPN	65			65(100)	16 (25)	79.3 ± 3.3	66	27.4 ± 4.9	3.0 (0.5)	2.9 ± 1.0	54/11/0	6.9 ± 1.9	37 (29-44)	No	7
							CA	65			65(100)	5 (8)	79.3 ± 4.1	60	27.9 ± 5.9	2.9 (0.6)	3.0 ±1.0	48/17/0	6.4 ± 2.0	46 (38-53)		
Bianchi, L (12).	2021	Survival, recurrence, complications, renal function	Int J Urol	Italy	R	2007-2019	MIPN	137	2.4 (2–3)				72 (62–77)	66.4	26 (24–29)		2.4 (2–3)	107/30/0		52 (32–99)	Yes (propensity score matching)	7
							Ablation	137	2.3 (1.8–2.9)				72 (65–79)	65.7	26 (24–28)		2.3 (1.8–2.9)	106/19/12		62 (47–79)		
Bird (13)	2009	Survival, recurrence, complications, renal function	Journal of Endourology	USA	R	2002-2007	LPN	33	20 (60.6)				57.8 (27–77)	55	28.45	2.2	3.1	.20/13/0	N/A	27 (6–58)	No	7
							LRFA	36	26 (72.3)				75.2 (56–86)	61	30.08	2.8	2.8	26/13/0		12 (6–23)		
Caputo (14)	2017	Survival, recurrence, complications	European Urology	USA	P	1999-2014	RAPN	31	28 (90)	8 (36.5)	31 (100)	30 (96.8)	61 (52–68)	67	30.6 (26.6–35.4)	3 (2–3)	5.0 (4.5–5.6)	28/3/0	9.0 (8–10)	13.0 (3.19–19.2)	Yes (propensity score matching)	8
							CA	31	22 (71)	12 (42.6)	31 (100)	27 (87.1)	68 (64–76)	81	30.6 (26.3–37.4)	3 (3–3)	4.3 (4.2–4.7)	.22/8/1	8.0 (6–9)	30.1 (13.2–64.0)		
Desai (15)	2005	Survival, recurrence, complications	Urology	USA	P	1999-2003	LPN	153					60.59 ± 13.19	58	29.06 ± 6.42	≥3(46)	2.25 ± 0.67	N/A	N/A	5.8 (1–36)	No	6
							LCA	89					65.55 ± 12.69	69	27.43 ± 5.59	≥3(75)	2.05 ± 0.56			24.6 (1–60)		
Emara (16)	2014	Survival, recurrence, complications, renal function	BJU international	UK	P	2010-2012	RAPN	47	33 (70)				60.5 (38–80)	80	N/A	N/A	3.278 ± 1.787	33/14/0	5.77 ± 0.25	16.50 ± 0.946	No	8
							CA	56	39 (70)				69.75 (42–90)	66			2.559 ± 0.958	.9/9/8	5.75 ± 0.23	31.30 ± 1.802		
Fossati (17)	2015	Survival, recurrence, complications, renal function	European urology focus	Italy	R	2000-2013	MIPN	206	153 (74)			194 (94)	60 (51–70)	69	26 (23–28)	≥3(17)	2.5 (2.0–3.4)	153/53/0	N/A	43	No	8
							LCA	166	105 (63)			136 (82)	66 (57–73)	73	25 (23–29)	≥3(30)	2.0 (1.5–2.5)	105/43/18		39		
Garcia, R. G (18).	2021	Recurrence, complications	CardioVascular and Interventional Radiology	Brazil	R	2008-2017	RAPN	69			69 (100)	2 (3)	54.8 ± 11.9	72.4	27.5 ± 3.8	≥3(0)	N/A	N/A	N/A	22.1	No	7
							PCA	63			63 (100)	0 (0)	62.5 ± 14.1	76.2	28.3 ± 4.5	≥3(24.5)				22.1		
Guillotreau (19)	2012	Recurrence, complications, renal function	European Urology	USA	R	1998-2010	RAPN	210	156 (74)			36 (17)	57.8 ± 11.8	58	30.1 ± 6.4	≥3(51)	2.4 ± 0.8	N/A	N/A	4.8 (1–7.9)	Yes (multivariable logistic regression for complications)	8
							LCA	226	181 (77)			19 (8)	67.4 ± 11.3	71	29.3 ± 6.2	≥3(80)	2.2 ± 0.9			44.5 (8.7–66.8)		
Haber (20)	2012	Survival, recurrence, complications, renal function	BJU international	USA	R	1998-2008	LPN	48			48 (100)		60.6 ± 13.7	52.1	30.1 ± 6.2	2.7 ± 0.5	3.2 ± 1.33	31/17/0	N/A	42.7 ± 30.8	No	8
							LCA	30			30 (100)		60.9 ± 11.4	73.3	31.5 ± 5.8	2.7 ± 0.8	2.6 ± 1.08	25/5/0		60.2 ± 46.3		
Haramis (21)	2012	Survival, recurrence, complications	Journal of Laparoendoscopic and Advanced Surgical Techniques	USA	R	2005-2008	LPN	92				10 (10.9)	58.8 (37–85)	60.8	N/A	N/A	1.9 (0.3–4.5)	N/A	N/A	21.8 (1–48)	No	6
							LCA	75				5 (0.07)	69.2 (19–84)	62.7		1.9 (1–3)	2.0 (0.4–7.5)			14 (1–34)		
Ji (22)	2016	Recurrence, complications	Urologia internationalis	China	R	2006-2015	LPN	74					57.3 (25–76)	55.4	N/A	1.7 (1–3)	2.9 (1.4–3.8)	103/2/0	N/A	2.2 (1.7–3.3)	No	6
							LRFA	105					64.2 (42–81)	62.9		2.3 (1–3)	2.2 (1.7–3.3)	71/3/0		78 (60–106)		
Kim (23)	2015	Survival, recurrence, complications, renal function	Asian journal of surgery	South Korea	R	2005-2011	RAPN	27					60.33 ± 15.61	70.4	25.9 ± 3.4	N/A	1.77 ± 0.96	24/3/0	6.5 ± 1.7	10.9 ± 7.0	Yes (propensity score matching)	6
							RFA	27					58.67 ± 11.60	81.5	26.6 ± 3.1		1.8 ± 0.81	.3/2/24	6.3 ± 1.6	16.7 ± 10.5		
Kiriluk (24)	2011	Complications, renal function	Journal of Endourology	USA	P	2002-2008	LPN	51	41 (80.3)			6 (11.8)	66.0 (23–83)	51	29.1 (18.2–24)	N/A	2.27 (0.80–5.10)	N/A	N/A	18.3 (13.0–26.8)	No	7
							LAT	51	26 (50.9)			12 (23.5)	65.7 (27–75)	51	30.0 (12.1–56.9)		2.35 (0.99–4.90)			27.9 (0.4–40.0)		
Lian (25)	2010	Recurrence, complications, renal function	Chinese journal of surgery	China	R	2005-2009	LPN	29					61 (55-68)	66	N/A	N/A	2.8 (2.0-4.5)	N/A	N/A	27 (3-36)	No	6
							LCA	18					63 (41-73)	78			2.9 (1.5-5.0)			16 (6-21)		
Link (26)	2006	Recurrence	Journal of Endourology	USA	R	2004-2005	LPN	217					N/A	N/A	N/A	N/A	2.6 ± 1.3	N/A	N/A	N/A	No	7
							LCA	28									2.4 ± 0.9					
Liu (27)	2021	Survival, recurrence, complications, renal function	Diagnostics	China	R	2008, 2015	RAPN	55	32 (58.2)		53 (96.4)	0 (0)	57.27 ± 13.28	52.7	25.29 ± 4.58	≥3(23.6)	4.06 ± 2.01	N/A	N/A	33.20 ± 19.55	Yes: matching	7
							LCA	55	27 (49.1)		54 (98.2)	3 (5.5)	59.44 ± 14.77	52.7	25.04 ± 4.23	≥3(20)	3.86 ± 2.13			54.96 ± 34.59		
O'Malley (28)	2007	Recurrence, complications	BJU International	USA	R	2003-2005	LPN	15					75.7 ± 4.6	79	27.1 ± 3.9	≥3(53)	2.5 ± 1.0	N/A	N/A	9.83 ± 8.8	Yes: matching	8
							LCA	15					76.1 ± 4.5	57	29.1 ± 6.8	≥3(62)	2.7 ± 1.3			11.9 ± 7.2		
Pantelidou (29)	2016	Recurrence, complications, renal function	CardioVascular and Interventional Radiology	UK	R	2005-2013	RAPN	63	59 (93.7)			10 (15.9)	54 ± 7	N/A	N/A	2 (2–3)	2.88 ± 0.13	63/0/0	7.38 ± 0.16	18.5 (6.2–29.5)	No	7
							RFA	63	63 (100)			4 (6.3)	61 ± 21			2 (2–3)	2.11 ± 0.19	59/0/4	7.38 ± 0.16	47.5 (11.8–80.2)		
Park (30)	2018	Survival, recurrence, complications	European radiology	Republic of Korea	R	2008-2016	RAPN	63	54 (85.7)			3 (4.8)	57.7 ± 10.8	75	N/A	1.8 ± 0.3	2.0 ± 0.6	63/0/0	7.1 ± 1.7	24.6 (1-90)	Yes: matching	8
							RFA	63	48 (76.2)			3 (4.8)	57.1 ± 13.1	65		1.8 ± 0.7	2.1 ± 0.5	63/0/0	7.2 ± 1.5	21 (1-65)		
Tanagho (31)	2013	Recurrence, complications, renal function	Journal of Endourology	USA	R	2007-2012	RAPN	233	80 (52.3)				57.4 ± 11.9	54.5	30.1 ± 6.0	N/A	2.9 ± 1.5	185/48/0	7.3 ± 1.9	21.9 ± 18.8	No	
						2000-2003	CA	267	185 (79.4)				69.3 ± 11.0	61	30.4 ± 7.8		2.5 ± 1.0	80/73/114	6.4 ± 1.7	39.8 ± 34.3		
Turna (32)	2009	Survival, recurrence, complications, renal function	The Journal of urology	USA	R	1997-2006	LPN	36	23 (63.8)				60.3 ± 15.5	58	30.5 ± 7.1	≥3(66.7)	3.7 ± 1.9	N/A	N/A	80	No	8
							RFA	36	22 (73.3)				64.1 ± 11.1	64	31.3 ± 5.7	≥3(77.7)	2.5 ± 1.1			80		
Uemura, T (33).	2021	Survival, recurrence, complications, renal function	In Vivo	Japan	R	2016-2019	RAPN	78	58 (74.3)		78 (100)	76 (97.4)	61 (52-69)	63	23 (21-25)	≥3(1.3)	1.9 (1.5-2.3)	N/A	≥10(3.8)	18.5 (12-30)	No	7
							PCA	48	41 (85.4)		48 (100)	47 (97.9)	78 (70-82)	41	23 (21-26)	≥3(33.3)	2.6 (2.0-3.4)		≥10(10.8)	12 (6-32)		
Yanagisawa (34)	2020	Survival, recurrence, complications, renal function	Urologic oncology	Japan	R	2011-2019	LPN	90	65 (72)		90 (100)	87 (96.7)	69.5 (63-75)	81	N/A	N/A	28.8 ± 9.5	88/0/2	6 (5-8)	18	Yes: matching	7
							PCA	90	88 (97.8)		90 (100)	89 (98.9)	68.5 (61−76)	76			27.6 ± 9.7	65/25/0	6 (5-7)	26.5		
Yu (35)	2020	Survival, recurrence, complications, renal function	Radiology	China	R	2006-2017	LPN	185			185 (100)		60.4 ± 14.1	74.6	N/A	N/A	2.3 ± 0.9	185/0/0	N/A	40.6 (25.1–63.4)	Yes: matching	8
							MWA	185			185 (100)		63.2 ± 15.2	74			2.3 ± 0.5	185/0/0		42.0 (23.5–69.3)		

Matching: 1 - Age; 2 - BMI; 3 - ASA; 4 - Charlson; 5 - Gender; 6 - Pathological stage; 7 - Urinary diversion type. RARC, robot-assisted partial nephrectomy; LPN, laparoscopic partial nephrectomy; RFA, Radiofrequency ablation; CRA, Cryoablation; MWA, Microwave ablation; RCT, randomized controlled trial; R, Retrospective; P, Prospective; NA, data not available; NOS, score; Newcastle-Ottawa Scale score.

### Statistical Analysis

Statistical analysis was performed by Review Manager, version 5.2 (The Cochrane Collaboration, Oxford, UK) Stata v.12.0 (Stata Corp LLC, College Station, TX, USA). For this meta-analysis, if the heterogeneity test was I^2^ > 50%, P < 0.1, then we used the random effect model; if the heterogeneity test was I^2^ < 50%, P > 0.1, then we used the fixed utility model. The combined r-values and 95% confidence intervals (CIs) of each study were calculated, and the forest map displayed the characteristics of each study result. The quality of the included literature was evaluated using the Newcastle–Ottawa scale (NOS). The Begg’s and Egger’s tests were used to test the publication bias. The P < 0.05 was indicated statistically significant.

## Results

### Eligible Studies and Study Characteristics

**Figure 1:**
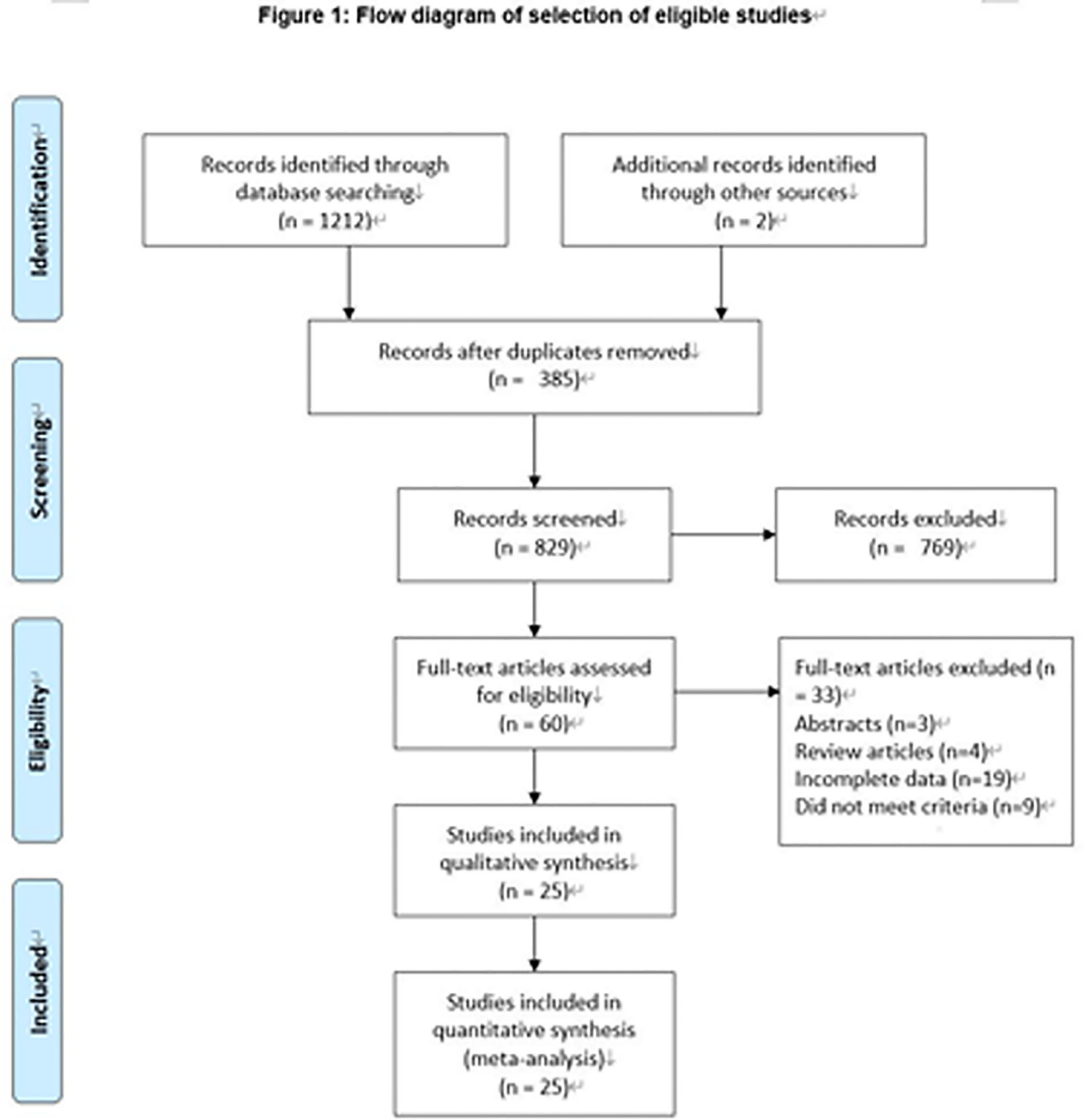
Flowchart for records selection process of the meta-analysis [According to PRISMA template: Moher D, Liberati A, Tetzlaff J, Altman DG, The PRISMA Group (2009). Preferred Reporting Items for Systematic Reviews and Meta-Analyses: The PRISMA Statement. PLoS Med 6(7): e1000097. doi:10.1371/journal. Pmed 1000097].

We initially searched 1,206 records. A total of 385 literature studies that were published repeatedly and cross-published were deleted. After reading the title and abstract, 760 articles were excluded. After the remaining 61 pieces of literature were searched for full text, reading, and quality assessment, 26 pieces of literature (10–35) (4,420 participants: MIPN: 2031 vs. FT: 2389) were eventually included (**Figure 1**). The detailed information of this literature was listed in **Table 1**.

### Perioperative Outcomes

**Figure 2:**
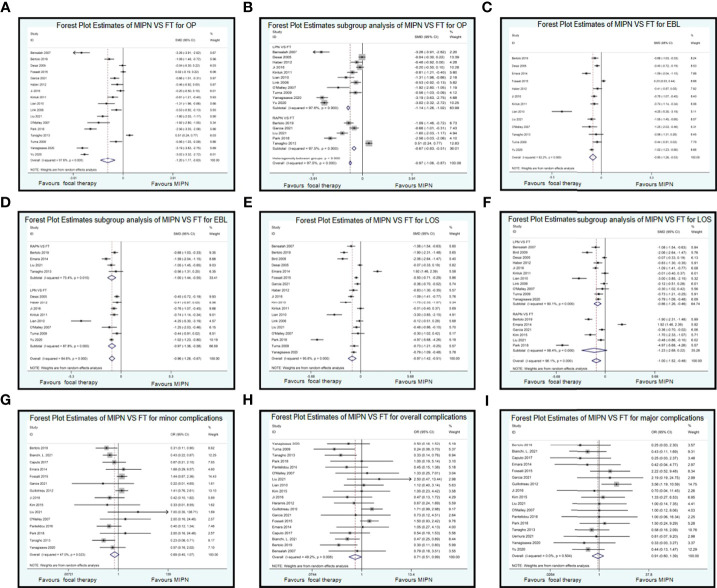
Forest plots of perioperative outcomes: perioperative outcomes. Forest Plot Estimates of MIPN VS FT for OP **(A)**, EBL **(C)**, LOS **(E)**, minor complications **(G)**, overall complications **(H)**, and major complications **(I)**. Forest Plot Estimates subgroup analysis of MIPN VS FT for OP **(B)**, EBL **(D)**, and LOS **(F)**. MIPN, minimally invasive partial nephrectomy; FT, focal therapy; OP, operating time; EBL, estimated blood loss; LOS, length of stay.

Data on OP were reported in 17 studies (10, 11, 15, 17, 18, 20, 22, 24–28, 30–32, 34, 35). Compared with MIPN, patients who underwent FT had significantly lower OP (SMD, −1.20; CI, −1.77 to −0.63; I^2^ = 97.6%, P < 0.0001) (**Figure 2A**). Owing to high heterogeneity (I^2^ = 95%), we chose subgroup analysis. Compared with FT, patients who underwent LPN had significantly higher OP (SMD, −1.14; CI, −1.26 to −1.02; I^2^ = 97.6%, P < 0.0001) and patients who underwent RAPN had significantly higher OP (SMD, −0.67; CI, −0.83 to −0.51; I^2^ = 97.5%, P < 0.0001) (**Figure 2B**). Sensitivity analysis and subgroup analysis cannot reduce heterogeneity.

We included 13 studies (11, 15–17, 20, 22, 24, 25, 27, 28, 31, 32, 35) about EBL. Compared with MIPN, patients who underwent FT had significantly less EBL (SMD, −0.90; CI, −1.26 to −0.53; I^2^ = 92.2%, P < 0.0001) (**Figure 2C**). Owing to high heterogeneity (I^2^ = 92.2%), we chose subgroup analysis. Compared with FT, patients who underwent LPN had significantly higher EBL (SMD, −0.97; CI, −1.36 to −0.58; I^2^ = 87.8%, P < 0.0001) and patients who underwent RAPN had significantly higher EBL (SMD, −1.00; CI, −1.44 to −0.55; I^2^ = 73.4%, P = 0.01) (**Figure 2D**). We subgroup analysis by nephropathy recently published 2022 back to 2017 (5 years) vs. older studies. There is only subgroup analysis by nephropathy recently published in 2022 back to 2017 (5 years) (SMD, −0.95; CI, −1.11 to −0.78; I^2^ = 26.8%, P = 0.247) vs. older studies (SMD, −0.44; CI, −0.56 to −0.32; I^2^ = 92.2%, P = 0.0001) difference here for estimated blood loss (EBL). Sensitivity analysis cannot reduce heterogeneity.

We included 18 studies (10, 11, 13, 15–18, 20, 22–28, 30, 32, 34) on LOS. Compared with MIPN, patients who underwent FT had significantly less LOS (SMD, −0.90; CI, −1.26 to −0.53; I^2^ = 92.2%, P < 0.0001) (**Figure 2E**). Owing to high heterogeneity (I^2^ = 92.2%), we chose subgroup analysis. Compared with FT, patients who underwent RAPN had significantly higher LOS (SMD, −0.86; CI, −1.26 to −0.46; I^2^ = 90.1%, P < 0.0001) and patients who underwent LPN had significantly higher LOS (SMD, −1.23; CI, −2.68 to 0.22; I^2^ = 98.4%, P < 0.0001) (**Figure 2F**). Sensitivity analysis cannot reduce heterogeneity.

Compared with MIPN, patients who underwent FT had significantly less minor complication (Clavien 1–2) (OR, 0.69; CI, 0.45 to 1.07; I^2^ = 47%, P = 0.023) (**Figure 2G**) and overall complication (OR, 0.71; CI, 0.51 to 0.99; I^2^ = 49.2%, P = 0.008) (**Figure 2H**). There is a similarity between MIPN and FT for major complications (Clavien 3–5) (OR, 0.91; CI, 0.60 to 1.39; I^2^ = 0%, P = 0.504) (**Figure 2I**).

### Renal Functional Outcomes

**Figure 3:**
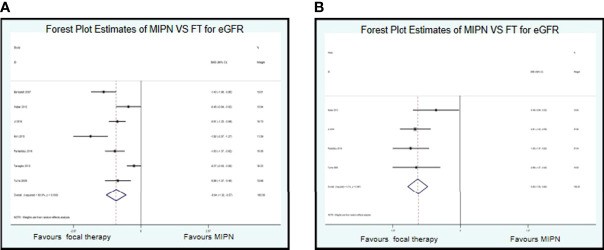
Forest plots of perioperative outcomes: renal functional outcomes. Forest Plot Estimates of MIPN VS FT for eGFR **(A)** (I2=83.9%) and **(B)** (I2=5.1%). MIPN, minimally invasive partial nephrectomy; FT, focal therapy.

For the functional results, we conducted a systematic analysis of eGFR. Data on eGFR were reported in seven studies. Compared with MIPN, patients who underwent FT had significantly reduced in eGFR (SMD, −0.94; CI, −1.32 to −0.57; I^2^ = 83.9%, P < 0.0001) (**Figure 3A**). Owing to high heterogeneity (I^2^ = 83.9%), we chose sensitivity analysis. After omitting the studies by Bensalah et al. (10), Kim et al. (23), and Tanagho et al. (31), as samples that were left out, the pooled results change substantially, but the heterogeneity was significantly reduced (SMD, −0.850; CI, −1.050 to −0.650; I^2^ = 5.1%, P = 0.367) (**Figure 3B**).

### Oncological Outcomes

**Figure 4:**
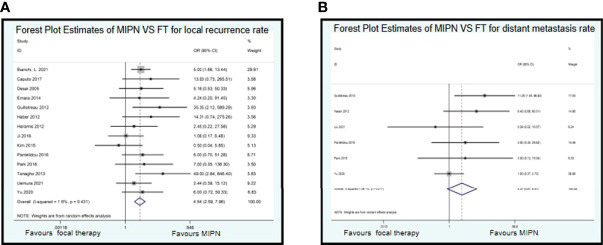
Forest plots of perioperative outcomes: renal functional outcomes. Forest Plot Estimates of MIPN VS FT for local recurrence rate **(A)** and distant metastasis rate **(B)**. MIPN, minimally invasive partial nephrectomy; FT, focal therapy.

The median or mean follow-up period of oncological outcomes of MIPN was 2.2 to 42.7 months, and FT was 14 to 78 months. Fourteen studies recorded on local recurrence rate, and six studies recorded on distant metastasis rate. There is a similarity between MIPN and FT for local recurrence rate (OR, 4.54; CI, 2.59 to 7.96; I^2^ = 1.6%, P = 0.431) (**Figure 4A**) and distant metastasis rate (OR, 2.37; CI, 0.87 to 6.47; I^2^ = 29.1%, P = 0.217) (**Figure 4B**).

### Publication Bias

We conducted publication bias on more than 15 included studies using Egger’s test. For OP, Egger’s test results revealed that t = −2.39, P = 0.051 in **Supplementary Figure 1A** and funnel plots in **Supplementary Figure 1B**. For LOS, Egger’s test results revealed that t = −1.73, P = 0.106 in **Supplementary Figure 1C** and funnel plots in **Supplementary Figure 1D**. For overall complication, Egger’s test results revealed that t = 1.11, P = 0.281 in **Supplementary Figure 1E** and funnel plots in **Supplementary Figure 1F**. For major complications (Clavien 3–5), Egger’s test results revealed that t = 0.97, P = 0.345 in **Supplementary Figure 1G** and funnel plots in **Supplementary Figure 1H**. There is no publication bias in the above.

## Discussion

In recent years, with the development of minimally invasive technology, SRMs were mainly treated by MIPN. For the clinical application of ablation technology, SRM ablation therapy has thus entered a new era (36, 37). The purpose of SRMs by MIPN or FT was to treat tumors while reducing perioperative complications, protecting the function of the kidney, and decreasing the postoperative recurrence rate (38). Therefore, the best treatment plan depends on the perioperative period, renal function, and tumor outcome. At present, there are few reports on the relationship between MIPN and FT.

MIPN has replaced the traditional radical nephrectomy with the increase of SRMs patients’ willingness to protect the kidney and the progress of corresponding surgical techniques in urology. A study has concluded that it is no statistically significant difference between MIPN and radical nephrectomy in terms of tumor control effects (39). At the same time, MIPN not only preserves the patient’s nephrons but also is minimally invasive. Therefore, MIPN has become the main treatment for SRMs and early renal cancer. However, because MIPN requires renal artery block during the operation, the long-term renal function damage caused by this has also become a deficiency of MIPN (40–42). There have always been controversies regarding the treatment of SRMs between FT and MIPN, but unfortunately, due to the shortcomings of retrospective research, the level of credibility of the relevant conclusions is not high. To our knowledge, this study provides a new systematic review and meta-analysis comparing MIPN and FT for SRMs. Because of the lack of RCTs, we have investigated 25 non-randomized observational studies comparing MIPN and FT. The primary endpoint is the oncology outcome (local tumor recurrence and distant metastasis). The secondary endpoints are renal function and perioperative outcomes. Because of the short follow-up period, the meta-analysis of CSS and OS is inappropriate. However, a multi-center retrospective analysis showed that RAPN has good long-term oncologic and functional outcomes of the procedure, which duplicate those achieved in historical series of laparoscopic surgery (43).

The meta-analysis emphasizes that FT has a disadvantage in Oncological control compared to MIPN. Compared with patients who received MIPN, patients who have received FT had an OR of 2.43 for distant metastases and an OR of 6.59 for local recurrence. For the reasons, on the one hand, the overall follow-up period of the oncologic outcomes in the FT group is long, which may lead to a relatively high recurrence rate, especially metastasis rate. The different follow-up periods between MIPN and FT affect both distant metastasis rate and local recurrence rate. In addition, considering the secondary efficacy, which is the confirmed oncologic outcomes after the second FT, the risk of recurrence can be reduced (44). In one study, compared with MIPN, secondary FT seemed to be effective for cancer control and the metastasis rate was not high (45). However, there is no second FT in the included literature. Interestingly, matching studies with similar basic characteristics showed no difference in local recurrence rates between MIPN and FT (23, 29, 35). On the other hand, the firing diameter of FT covers the tumor edge 0.5–1 cm (46). In some anomalistic tumors, FT cannot guarantee complete coverage of the entire tumor. MIPN only needs to ensure that the tumor capsule is intact. Several studies have confirmed that MIPN is more effective in local recurrence rate and distant metastasis rate compared with FT (20, 35).

Conversely, for the meta-analysis results, we mainly describe the perioperative outcomes of MIPN and FT in SRMs. Patients who underwent FT had significantly lower OP of MD (60.34 min), lower EBL of MD (50.28 ml), and LOS of MD (1.95 day) compared with MIPN. For the renal functional outcomes, patients who underwent FT had significantly lower eGFR of WD (8.56 ml/min/1.73m^2^) compared with MIPN. There are two main reasons that FT has a lower OP, BEL, LOS, and eGFR than MIPN. On the one hand, FT has the advantages of convenient operation and small trauma (47). Research by Park et al. also confirmed that FT has the above results (30). On the other hand, compared with MIPN, FT does not need to block the renal artery, thereby reducing the renal warm ischemia time and ischemia-reperfusion injury and further preserving the advantages of renal function. A system analysis study also confirmed this view (44). Moreover, the EAU guidelines suggest that FT is feasible for renal insufficiency or isolated renal tumors (7). However, there is only subgroup analysis by nephropathy recently published in 2022 back to 2017 (5 years) vs. older studies difference here for EBL. The possible reason is that with the improvement of minimally invasive surgical techniques, the amount of surgical bleeding in 2022 back to 2017 has been significantly controlled. Therefore, compared with FT, there was no difference in the amount of EBL. In addition these are also explained in the discussion section of the article.

We used postoperative complication graded by Clavien Dindo classification for complication analysis (48). Interestingly, no matter one of the minor complications (Clavien 1–2), major complications (Clavien 3–5), and overall complications are similar between MIPN and FT. MIPN has a higher complication rate compared with FT in many studies (31, 35). Although the management of SRMs by MIPN has developed rapidly, the incidence of major complications is still higher than that of FT but not statistically significant. However, all overall complication rates, max complication rates, and minor complication rates in the FT group and MIPN group were lower. Moreover, it did not reach statistical significance.

Choosing MIPN or FT requires comprehensive consideration of the patient’s underlying disease, tumor characteristics (size, number, and anatomical relationship), kidney disease stage, life expectancy, comorbidities, and other related factors (49, 50). The diameter of tumors reported in literature studies is about 2–3 cm, and a few studies are up to 7 cm in size (13, 51). Compared with MIPN, patients receiving FT have smaller average tumor size, relatively uncomplicated anatomical location, multiple renal tumors, endogenous tumors, and other factors (52, 53). Among the SRMs patients treated with FT, most of the patients’ R.E.N.A.L nephrectomy scores showed that the complexity of the operation was Low or medium (11, 34). In adition, part of the literature included in this meta-analysis uses renal measurement scores to assess the complexity of surgery also confirms the appeal argument (11, 14, 16, 23, 29–31, 34). Therefore, we are considering whether to choose MIPN or FT treatment. The Charlson comorbidity index and tumor complexity score can replace the age and tumor size reference factors to make the most profitable decision (54).

Our meta-analysis has several limitations. First, none of the literature in this meta-analysis is a randomized controlled trial. Second, the meta-analysis mainly recorded observational studies and may be affected by factors such as bias and confounding. Third, the OP and LOS in this article are highly heterogeneous. The heterogeneity could not be ruled out after sensitivity analysis and subgroup analysis, so the random-effects model was selected. Fourth, we did not distinguish the surgical approach (laparoscopic vs. percutaneous vs. robotic). Moreover, the long-term prognosis of tumors cannot be fully assessed due to the lack of long-term and follow-up control studies of large cases.

## Conclusion

FT has more advantages in protecting kidney function, reducing bleeding, shortening operating time, and shortening the length of stay. There is no difference in local recurrence, distant metastasis, and major complications. For the minimally invasive era, we need to weigh the advantages and disadvantages of all aspects to make comprehensive choices.

## Data Availability Statement

The datasets presented in this study can be found in online repositories. The names of the repository/repositories and accession number(s) can be found in the article/**Supplementary Material**.

## Author Contributions

Conceptualization: LD and LiY. Data curation: LD, LuY, WLL, and WQ. Formal analysis: LD and LuY. Funding acquisition: LD. Methodology: LiY. Investigation: LD and LuY. Resources: WLL. Writing—original draft: WLL. Writing—review and editing: WQ. All authors contributed to the article and approved the submitted version.

## Funding

This work was supported by the Scientific Research Foundation of Health and Family Planning Commission of Sichuan Province (20PJ236).

## Conflict of Interest

The authors declare that the research was conducted in the absence of any commercial or financial relationships that could be construed as a potential conflict of interest.

## Publisher’s Note

All claims expressed in this article are solely those of the authors and do not necessarily represent those of their affiliated organizations, or those of the publisher, the editors and the reviewers. Any product that may be evaluated in this article, or claim that may be made by its manufacturer, is not guaranteed or endorsed by the publisher.

## Supplementary Material

Supplementary Figure 1 Egger’s publication bias plot to detect publication bias and Funnel plot to detect publication bias.

## Abbreviations

MIPN, minimally invasive partial nephrectomy; FT, focal therapy; SRMs, small renal masses; RFA, radiofrequency ablation; MWA, microwave ablation; CA, cryoablation; IRE, irreversible electroporation; SBRT, stereotactic body radiation therapy; MD, mean difference; ORs, odds ratios; CIs, confidence intervals; AUA, American Urological Association; EAU, European Association of Urology; RAPN, robot-assisted partial nephrectomy; LPN, laparoscopic partial nephrectomy; PRISMA, Preferred Reporting Items for Systematic Reviews and Meta-Analysis; NOS, Newcastle–Ottawa scale; OP, operating time; EBL, estimated blood loss; LOS, length of stay.

## References

[1] Bray F Ferlay J Soerjomataram I . Global Cancer Statistics 2018: GLOBOCAN Estimates of Incidence and Mortality Worldwide for 36 Cancers in 185 Countries. CA: Cancer J Clin (2018) 68:394–424. doi: 10.3322/caac.21492 30207593

[2] Canil C Kapoor A Basappa NS Bjarnason G Bossé D Dudani S . Management of Advanced Kidney Cancer: Kidney Cancer Research Network of Canada (KCRNC) Consensus Update 2021. Can Urol Assoc J (2021) 15:84–97. doi: 10.5489/cuaj.7245 33830005 PMC8021420

[3] Abu-Ghanem Y Fernández-Pello S Bex A Ljungberg B Albiges L Dabestani S . Limitations of Available Studies Prevent Reliable Comparison Between Tumour Ablation and Partial Nephrectomy for Patients With Localised Renal Masses: A Systematic Review From the European Association of Urology Renal Cell Cancer Guideline Panel. Eur Urol Oncol (2020) 3:433–52. doi: 10.1016/j.euo.2020.02.001 32245655

[4] Sanchez A Feldman AS Hakimi AA . Current Management of Small Renal Masses, Including Patient Selection, Renal Tumor Biopsy, Active Surveillance, and Thermal Ablation. J Clin Oncol (2018) 36:3591–600. doi: 10.1200/JCO.2018.79.2341 PMC6804853 30372390

[5] Johnson BA Cadeddu JA . Current Opinion in Urology 2017: Focal Therapy of Small Renal Lesions. Curr Opin Urol (2018) 28:166–71. doi: 10.1097/MOU.0000000000000475 29303914

[6] Campbell S Uzzo RG Allaf ME Bass EB Cadeddu JA Chang A . Renal Mass and Localized Renal Cancer: AUA Guideline. J Urol (2017) 198:520–9. doi: 10.1016/j.juro.2017.04.100 28479239

[7] Ljungberg B Bensalah K Canfield S Dabestani S Hofmann F Hora M . EAU Guidelines on Renal Cell Carcinoma: 2014 Update. Eur Urol (2015) 67:913–24. doi: 10.1016/j.eururo.2015.01.005 25616710

[8] Chan VW Abul A Osman FH Ng HH Wang K Yuan Y . Ablative Therapies Versus Partial Nephrectomy for Small Renal Masses - A Systematic Review and Meta-Analysis. Int J Surg (2022) 97:106194. doi: 10.1016/j.ijsu.2021.106194 34958968

[9] Liberati A Altman DG Tetzlaff J Mulrow C Gøtzsche PC Ioannidis JP . The PRISMA Statement for Reporting Systematic Reviews and Meta-Analyses of Studies That Evaluate Health Care Interventions: Explanation and Elaboration. PloS Med (2009) 6:e1000100. doi: 10.1371/journal.pmed.1000100 19621070 PMC2707010

[10] Bensalah K Zeltser I Tuncel A Cadeddu J Lotan Y . Evaluation of Costs and Morbidity Associated With Laparoscopic Radiofrequency Ablation and Laparoscopic Partial Nephrectomy for Treating Small Renal Tumours. BJU Int (2008) 101:467–71. doi: 10.1111/j.1464-410X.2007.07276.x 17922853

[11] Bertolo R Garisto J Armanyous S Agudelo J Lioudis M Kaouk J . Perioperative, Oncological and Functional Outcomes After Robotic Partial Nephrectomy vs. Cryoablation in the Elderly: A Propensity Score Matched Analysis. Urol Oncol: Semin Original Investigat (2019) 37:294.e9–294.e15. doi: 10.1016/j.urolonc.2018.12.016 30691958

[12] Bianchi L Chessa F Piazza P Ercolino A Mottaran A Recenti D . Percutaneous Ablation or Minimally Invasive Partial Nephrectomy for Ct1a Renal Masses? A Propensity Score-Matched Analysis. Int J Urol (2021) 29:222–8. doi: 10.1111/iju.14758 34894001

[13] Bird VG Carey RI Ayyathurai R Bird Y . Management of Renal Masses With Laparoscopic-Guided Radiofrequency Ablation Versus Laparoscopic Partial Nephrectomy. J Endourol (2009) 23:81–8. doi: 10.1089/end.2008.0087 19118475

[14] Caputo PA Zargar H Ramirez D Andrade HS Akca O Gao T . Cryoablation Versus Partial Nephrectomy for Clinical T1b Renal Tumors: A Matched Group Comparative Analysis. Eur Urol (2017) 71:111–7. doi: 10.1016/j.eururo.2016.08.039 27568064

[15] Desai MM Aron M Gill IS . Laparoscopic Partial Nephrectomy Versus Laparoscopic Cryoablation for the Small Renal Tumor. Urology (2005) 66:23–8. doi: 10.1016/j.urology.2005.06.114 16194703

[16] Emara AM Kommu SS Hindley RG Hindley N Barber J . Robot-Assisted Partial Nephrectomy vs Laparoscopic Cryoablation for the Small Renal Mass: Redefining the Minimally Invasive 'Gold Standard'. BJU Int (2014) 113:92–9. doi: 10.1111/bju.12252 24053473

[17] Fossati N Larcher A Gadda GM Sjoberg DD Mistretta FA Dell'Oglio P . Minimally Invasive Partial Nephrectomy Versus Laparoscopic Cryoablation for Patients Newly Diagnosed With a Single Small Renal Mass. Eur Urol Focus (2015) 1:66–72. doi: 10.1016/j.euf.2015.02.002 28723359

[18] Garcia RG Katz M Falsarella PM Malheiros DT Fukumoto H Lemos GC . Percutaneous Cryoablation Versus Robot-Assisted Partial Nephrectomy of Renal T1A Tumors: A Single-Center Retrospective Cost-Effectiveness Analysis. Cardiovasc Intervent Radiol (2021) 44(6):892–900. doi: 10.1007/s00270-020-02732-x 33388867

[19] Guillotreau J Haber GP Autorino R Miocinovic R Hillyer S Hernandez A . Robotic Partial Nephrectomy Versus Laparoscopic Cryoablation for the Small Renal Mass. Eur Urol (2012) 61:899–904. doi: 10.1016/j.eururo.2012.01.007 22264680

[20] Haber G-P Lee MC Crouzet S Kamoi K Gill IS . Tumour in Solitary Kidney: Laparoscopic Partial Nephrectomy vs Laparoscopic Cryoablation. BJU Int (2012) 109:118–24. doi: 10.1111/j.1464-410X.2011.10287.x 21895929

[21] Haramis G Graversen JA Mues AC Korets R Rosales JC Okhunov Z . Retrospective Comparison of Laparoscopic Partial Nephrectomy Versus Laparoscopic Renal Cryoablation for Small (<3.5 Cm) Cortical Renal Masses. J Laparoendosc Adv Surg Tech (2012) 22:152–7. doi: 10.1089/lap.2011.0246 22145574

[22] Ji C Zhao X Zhang S Liu G Li X Zhang G . Laparoscopic Radiofrequency Ablation Versus Partial Nephrectomy for Ct1a Renal Tumors: Long-Term Outcome of 179 Patients. Urol Internationalis (2016) 96:345–53. doi: 10.1159/000443672 26780439

[23] Kim SH Lee E-S Kim HH Kwak C Ku JH Lee SE . A Propensity-Matched Comparison of Perioperative Complications and of Chronic Kidney Disease Between Robot-Assisted Laparoscopic Partial Nephrectomy and Radiofrequency Ablative Therapy. Asian J Surg (2015) 38:126–33. doi: 10.1016/j.asjsur.2014.09.005 25458737

[24] Kiriluk KJ Shikanov SA Steinberg GD Shalhav AL Lifshitz DA . Laparoscopic Partial Nephrectomy Versus Laparoscopic Ablative Therapy: A Comparison of Surgical and Functional Outcomes in a Matched Control Study. J Endourol (2011) 25:1867–72. doi: 10.1089/end.2011.0087 21902540

[25] Lian H-b Guo H-q Gan W-d Li X.-g Yan X Zha S-w . A Retrospective Study Comparing the Clinical Efficacy of Laparoscopic Cryoablation and Partial Nephrectomy for Renal Cell Carcinoma. Zhonghua Wai Ke Za Zhi [Chinese J Surgery] (2010) 48:834–7. 21163052

[26] Link RE Permpongkosol S Gupta A Jarrett TW Solomon SB Kavoussi LR . Cost Analysis of Open, Laparoscopic, and Percutaneous Treatment Options for Nephron-Sparing Surgery. J Endourol (2006) 20:782–9. doi: 10.1089/end.2006.20.782 17094755

[27] Liu HY Kang CH Wang HJ Chen CH Luo HL Chen YT . Comparison of Robot-Assisted Laparoscopic Partial Nephrectomy With Laparoscopic Cryoablation in the Treatment of Localised Renal Tumours: A Propensity Score-Matched Comparison of Long-Term Outcomes. Diagnostics (2021) 11(5):759. doi: 10.3390/diagnostics11050759 33922727 PMC8146293

[28] O'Malley RL Berger AD Kanofsky JA Phillips CK Stifelman M Taneja SS . A Matched-Cohort Comparison of Laparoscopic Cryoablation and Laparoscopic Partial Nephrectomy for Treating Renal Masses. BJU Int (2007) 99:395–8. doi: 10.1111/j.1464-410X.2006.06554.x 17092288

[29] Pantelidou M Challacombe B McGrath A Brown M Ilyas S Katsanos K . Percutaneous Radiofrequency Ablation Versus Robotic-Assisted Partial Nephrectomy for the Treatment of Small Renal Cell Carcinoma. Cardiovasc Intervent Radiol (2016) 39:1595–603. doi: 10.1007/s00270-016-1417-z PMC5052326 27435582

[30] Park BK Gong IH Kang MY Sung HH Jeon HG Jeong BC . RFA Versus Robotic Partial Nephrectomy for T1a Renal Cell Carcinoma: A Propensity Score-Matched Comparison of Mid-Term Outcome. Eur Radiol (2018) 28:2979–85. doi: 10.1007/s00330-018-5305-6 29426988

[31] Tanagho YS Bhayani SB Kim EH Figenshau RS . Renal Cryoablation Versus Robot-Assisted Partial Nephrectomy: Washington University Long-Term Experience. J Endourol (2013) 27:1477–86. doi: 10.1089/end.2013.0192 24283518

[32] Turna B Kaouk JH Frota R Stein RJ Kamoi K Gill IS . Minimally Invasive Nephron Sparing Management for Renal Tumors in Solitary Kidneys. J Urol (2009) 182:2150–7. doi: 10.1016/j.juro.2009.07.066 19758655

[33] Uemura T Kato T Nagahara A Kawashima A Hatano K Ujike T . Therapeutic and Clinical Outcomes of Robot-Assisted Partial Nephrectomy Versus Cryoablation for T1 Renal Cell Carcinoma. In Vivo (2021) 35:1573–9. doi: 10.21873/invivo.12413 PMC8193303 33910838

[34] Yanagisawa T Miki J Shimizu K Fukuokaya W Urabe F Mori K . Functional and Oncological Outcome of Percutaneous Cryoablation Versus Laparoscopic Partial Nephrectomy for Clinical T1 Renal Tumors: A Propensity Score-Matched Analysis. Urol Oncol (2020) 38:938.e1–7. doi: 10.1016/j.urolonc.2020.09.024 33036899

[35] Yu J Zhang X Liu H Zhang R Yu X Cheng Z . Percutaneous Microwave Ablation Versus Laparoscopic Partial Nephrectomy for Ct1a Renal Cell Carcinoma: A Propensity-Matched Cohort Study of 1955 Patients. Radiology (2020) 294:698–706. doi: 10.1148/radiol.2020190919 31961239

[36] Volpe A Cadeddu JA Cestari A Gill IS Jewett MA Joniau S . Contemporary Management of Small Renal Masses. Eur Urol (2011) 60:501–15. doi: 10.1016/j.eururo.2011.05.044 21664040

[37] Withington J Neves JB Barod R . Surgical and Minimally Invasive Therapies for the Management of the Small Renal Mass. Curr Urol Rep (2017) 18:61. doi: 10.1007/s11934-017-0705-8 28664237

[38] Kimura M Baba S Polascik TJ . Minimally Invasive Surgery Using Ablative Modalities for the Localized Renal Mass. Int J Urol (2010) 17:215–27. doi: 10.1111/j.1442-2042.2009.02445.x 20070411

[39] Stewart SB Thompson RH Psutka SP Cheville JC Lohse CM Boorjian SA . Evaluation of the National Comprehensive Cancer Network and American Urological Association Renal Cell Carcinoma Surveillance Guidelines. J Clin Oncol (2014) 32:4059–65. doi: 10.1200/JCO.2014.56.5416 PMC4265116 25403213

[40] Faddegon S Ju T Olweny EO Liu Z Han WK Yin G . A Comparison of Long Term Renal Functional Outcomes Following Partial Nephrectomy and Radiofrequency Ablation. Can J Urol (2013) 20:6785–9. 23783048

[41] Olweny EO Park SK Tan YK Best SL Trimmer C Cadeddu JA . Radiofrequency Ablation Versus Partial Nephrectomy in Patients With Solitary Clinical T1a Renal Cell Carcinoma: Comparable Oncologic Outcomes at a Minimum of 5 Years of Follow-Up. Eur Urol (2012) 61:1156–61. doi: 10.1016/j.eururo.2012.01.001 22257424

[42] Nakamura A Osonoi T Terauchi Y . Relationship Between Urinary Sodium Excretion and Pioglitazone-Induced Edema. J Diabetes Investig (2010) 1:208–11. doi: 10.1111/j.2040-1124.2010.00046.x PMC4020723 24843434

[43] Carbonara U Simone G Capitanio U Minervini A Fiori C Larcher A . Robot-Assisted Partial Nephrectomy: 7-Year Outcomes. Minerva Urol Nephrol (2021) 73:540–3. doi: 10.23736/S2724-6051.20.04151-X 33200907

[44] Yoon YE Lee HH Kim KH Park SY Moon HS Lee SR . Focal Therapy Versus Robot-Assisted Partial Nephrectomy in the Management of Clinical T1 Renal Masses: A Systematic Review and Meta-Analysis. Med (Baltimore) (2018) 97:e13102. doi: 10.1097/MD.0000000000013102 PMC6250551 30407321

[45] Zangiacomo RN Martins GLP Viana PCC Horvat N Arap MA Nahas WC . Percutaneous Thermoablation of Small Renal Masses (T1a) in Surgical Candidate Patients: Oncologic Outcomes. Eur Radiol (2021) 31:5370–8. doi: 10.1007/s00330-020-07496-z 33392662

[46] Stein RJ Kaouk JH . Renal Cryotherapy: A Detailed Review Including a 5-Year Follow-Up. BJU Int (2007) 99:1265–70. doi: 10.1111/j.1464-410X.2007.06816.x 17441921

[47] Carbonara U Lee J Crocerossa F Veccia A Hampton LJ Eun D . Single Overnight Stay After Robot-Assisted Partial Nephrectomy: A Bi-Center Experience. Minerva Urol Nefrol (2020). doi: 10.23736/S0393-2249.20.04054-0 33200901

[48] Clavien PA Barkun J de Oliveira ML Vauthey JN Dindo D Schulick RD . The Clavien-Dindo Classification of Surgical Complications: Five-Year Experience. Ann Surg (2009) 250:187–96. doi: 10.1097/SLA.0b013e3181b13ca2 19638912

[49] Breau RH Crispen PL Jenkins SM Blute ML Leibovich BC . Treatment of Patients With Small Renal Masses: A Survey of the American Urological Association. J Urol (2011) 185:407–13. doi: 10.1016/j.juro.2010.09.092 21168170

[50] Millman AL Pace KT Ordon M Lee JY . Surgeon-Specific Factors Affecting Treatment Decisions Among Canadian Urologists in the Management of Pt1a Renal Tumours. Can Urol Assoc J (2014) 8:183–9. doi: 10.5489/cuaj.1884 PMC4081248 25024788

[51] Georgiades CS Rodriguez R . Efficacy and Safety of Percutaneous Cryoablation for Stage 1A/B Renal Cell Carcinoma: Results of a Prospective, Single-Arm, 5-Year Study. Cardiovasc Intervent Radiol (2014) 37:1494–9. doi: 10.1007/s00270-013-0831-8 24385225

[52] Klatte T Grubmüller B Waldert M Weibl P Remzi M . Laparoscopic Cryoablation Versus Partial Nephrectomy for the Treatment of Small Renal Masses: Systematic Review and Cumulative Analysis of Observational Studies. Eur Urol (2011) 60:435–43. doi: 10.1016/j.eururo.2011.05.002 21616582

[53] Kunkle DA Egleston BL Uzzo RG . Excise, Ablate or Observe: The Small Renal Mass Dilemma–A Meta-Analysis and Review. J Urol (2008) 179:1227–33; discussion 1233-4. doi: 10.1016/j.juro.2007.11.047 18280512

[54] Zondervan PJ van Lienden KP van Delden OM de laRosette JJ Laguna MP . Preoperative Decision Making for Nephron-Sparing Procedure in the Renal Mass: Time for Using Standard Tools? J Endourol (2016) 30:128–34. doi: 10.1089/end.2015.0472 26413831

