# Supplementary figures and images for: A Systematic Review and Meta-Analysis of Minimally Invasive Partial Nephrectomy Versus Focal Therapy for Small Renal Masses
Source: Front Oncol. 2022 May 26;12:732714. doi: 10.3389/fonc.2022.732714 (PMC9178090; doi:10.3389/fonc.2022.732714)

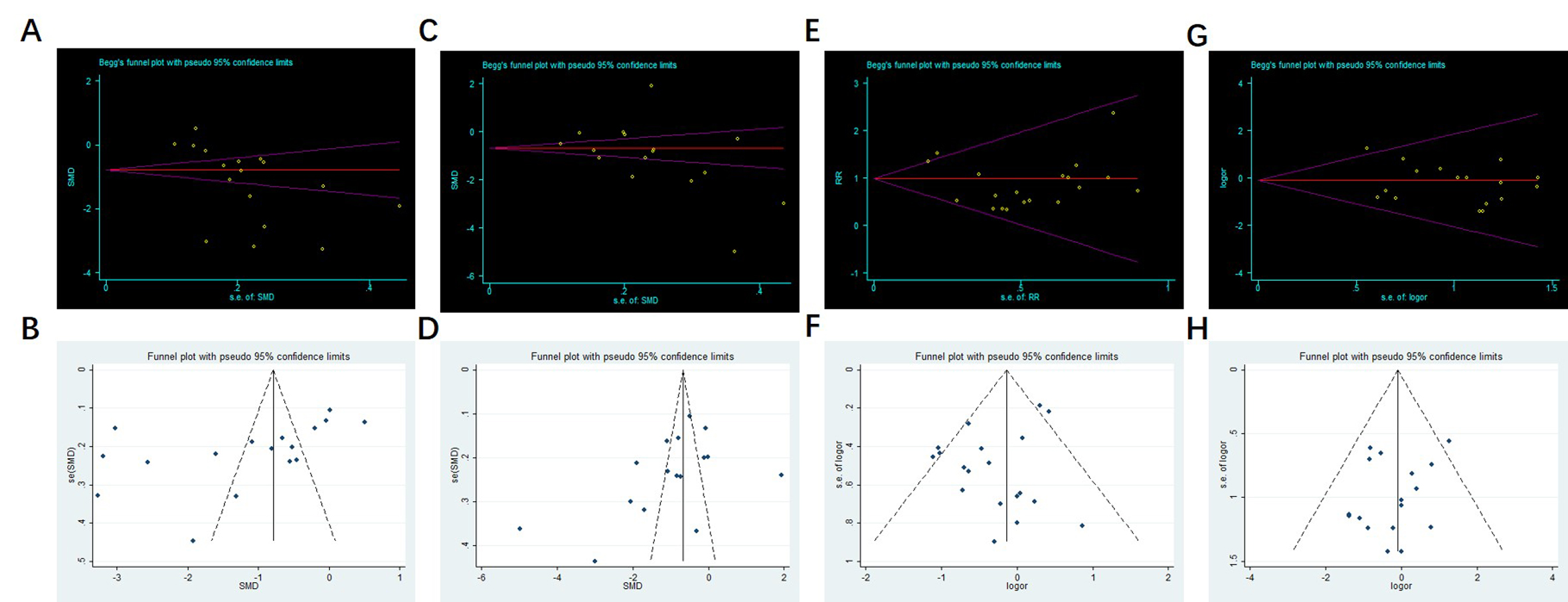

Supplement: Supplementary Figure 1 — Egger’s publication bias plot to detect publication bias and Funnel plot to detect publication bias. [file Image_1.jpeg]
