# Supplementary material for: A Systematic Review and Meta-Analysis of Minimally Invasive Partial Nephrectomy Versus Focal Therapy for Small Renal Masses
Source: Front Oncol. 2022 May 26;12:732714. doi: 10.3389/fonc.2022.732714 (PMC9178090; doi:10.3389/fonc.2022.732714)

Thank you very much for your advice.

We subgroup analysis by nephropathy scores >8 and nephropathy scores ≤ 8.

a) Table 1, includes articles that clearly describe nephropathy scores. we can see that there are only major complications (Clavien 3–5) and local recurrence.

major complications:

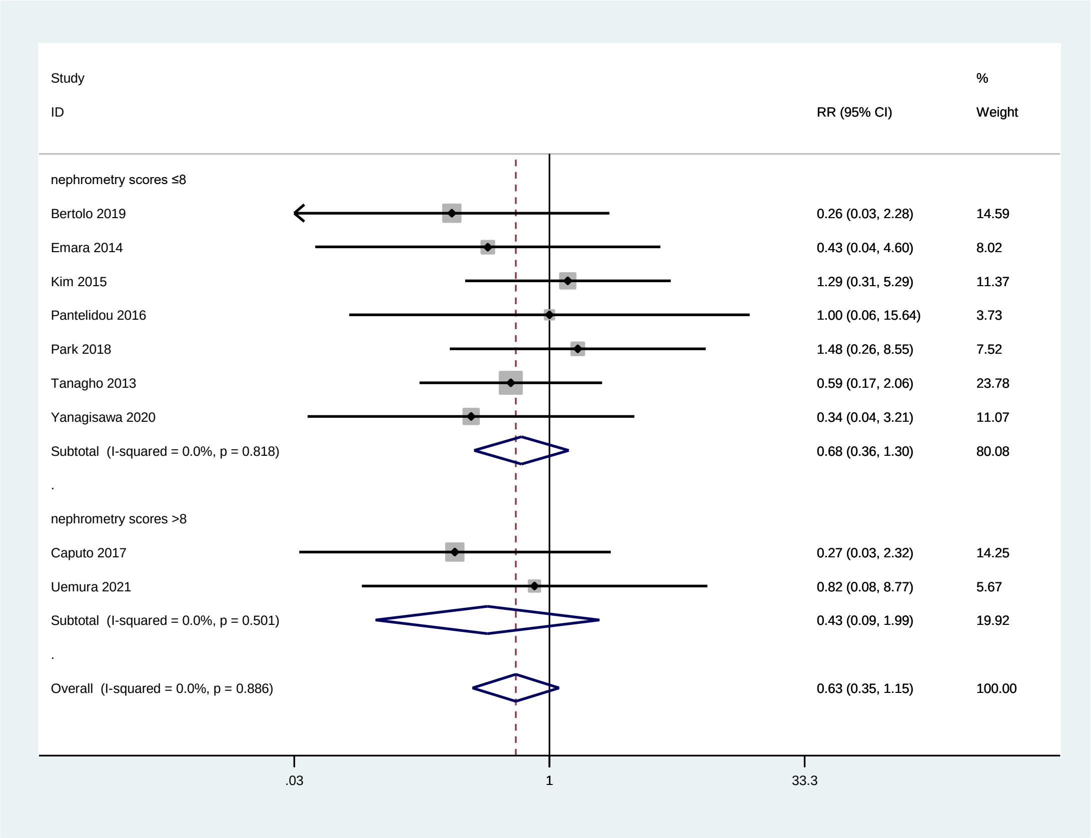

local recurrence:

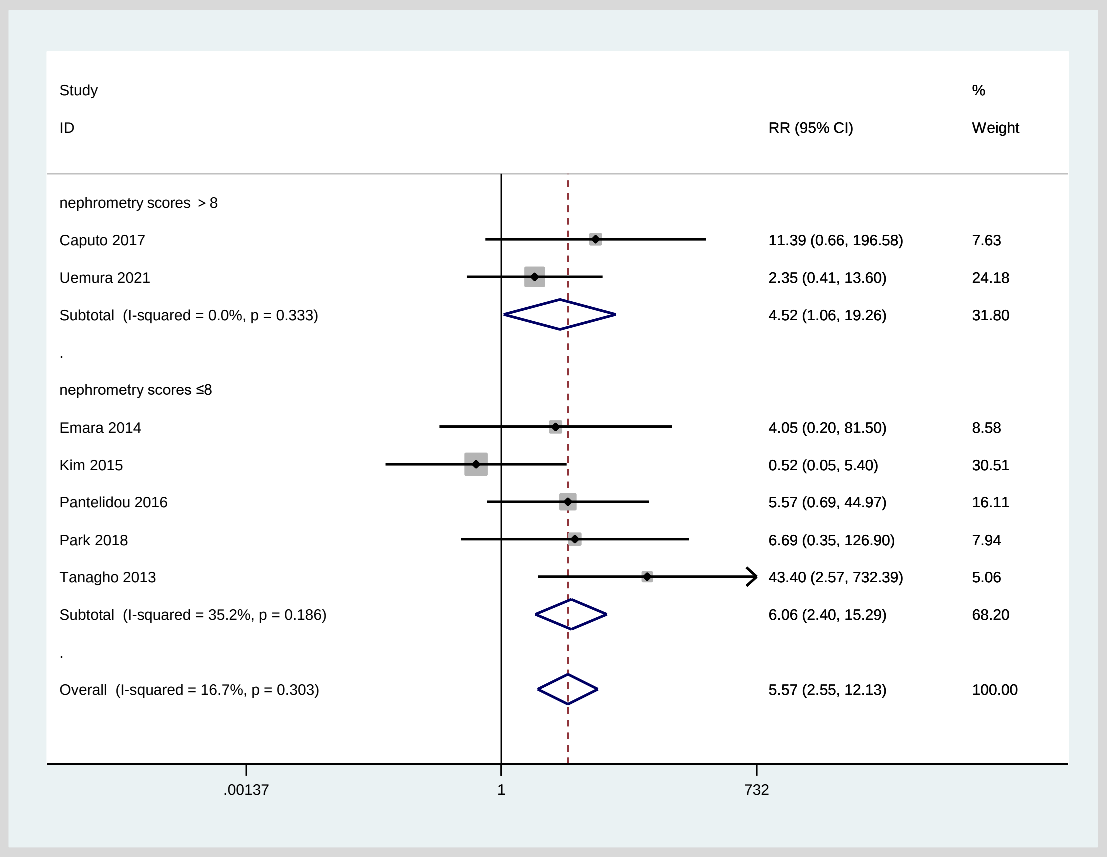

b) We subgroup analysis by nephropathy recently published 2022 back to 2017 (5 years) vs older studies. There is only subgroup analysis by nephropathy recently published 2022 back to 2017 (5 years) vs older studies difference here for Estimated blood loss. The possible reason is that with the improvement of minimally invasive surgical techniques, the amount of surgical bleeding in 2022 back to 2017 has been significantly controlled. Therefore, compared with FT, there was no difference in the amount of estimated blood loss. And these are also explained in the discussion section of the article. Other studies found no differences.

Operating time (OP):

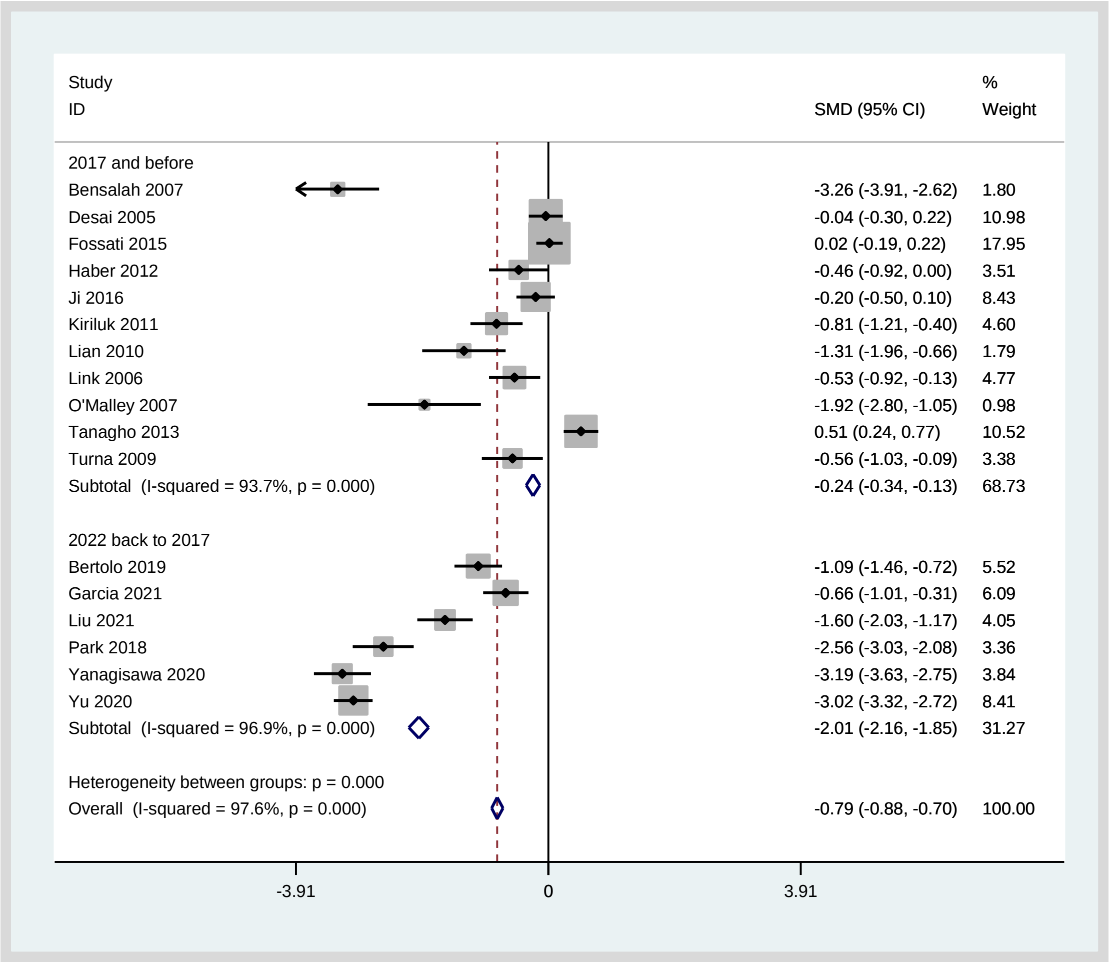

Estimated blood loss (EBL):

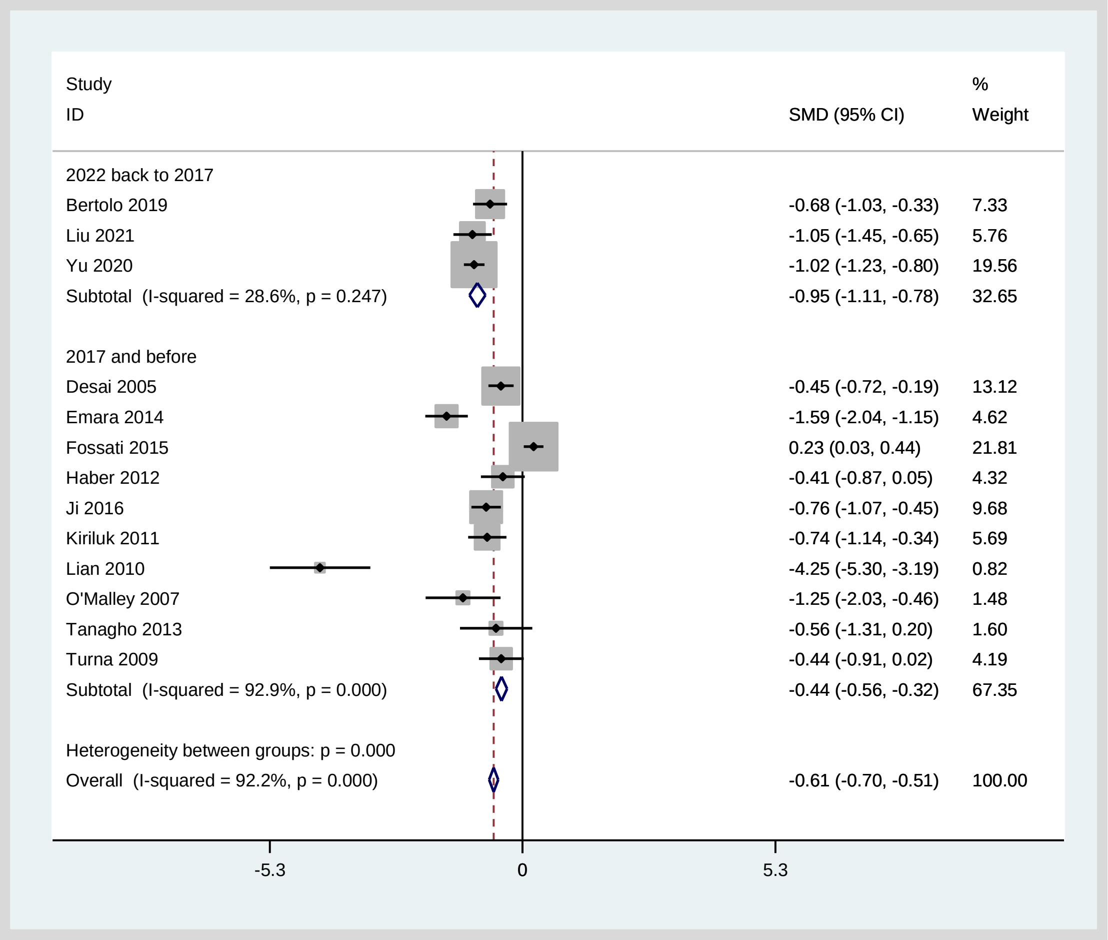

Length of stay (LOS):

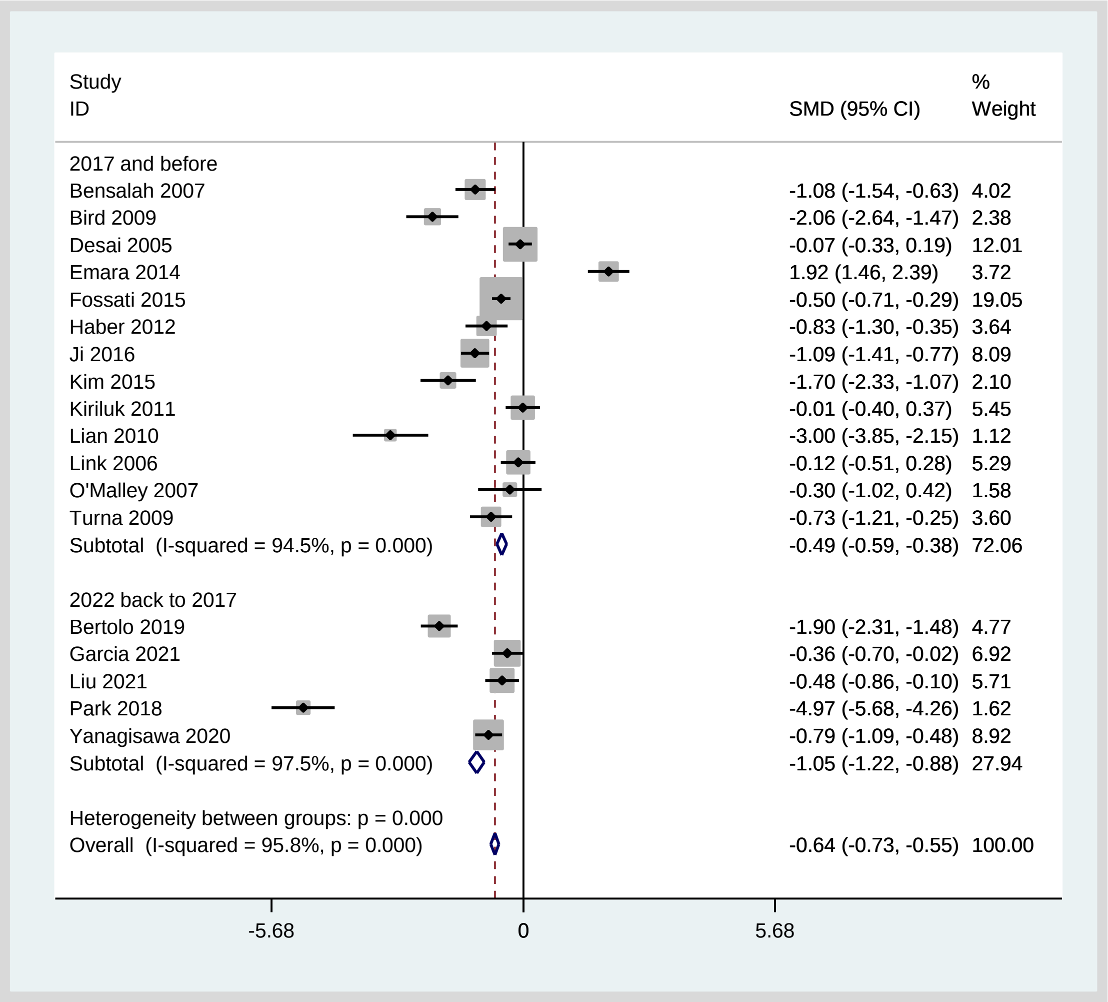

Distant metastasis:

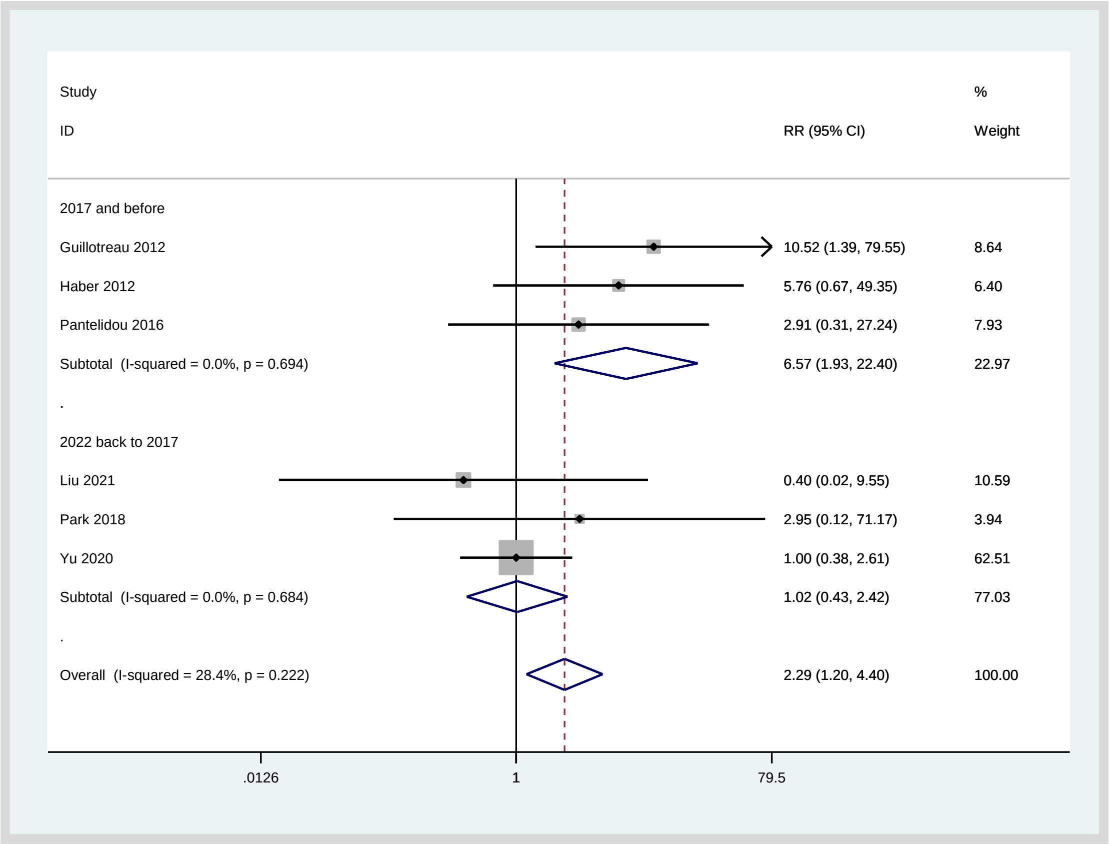

local recurrence:

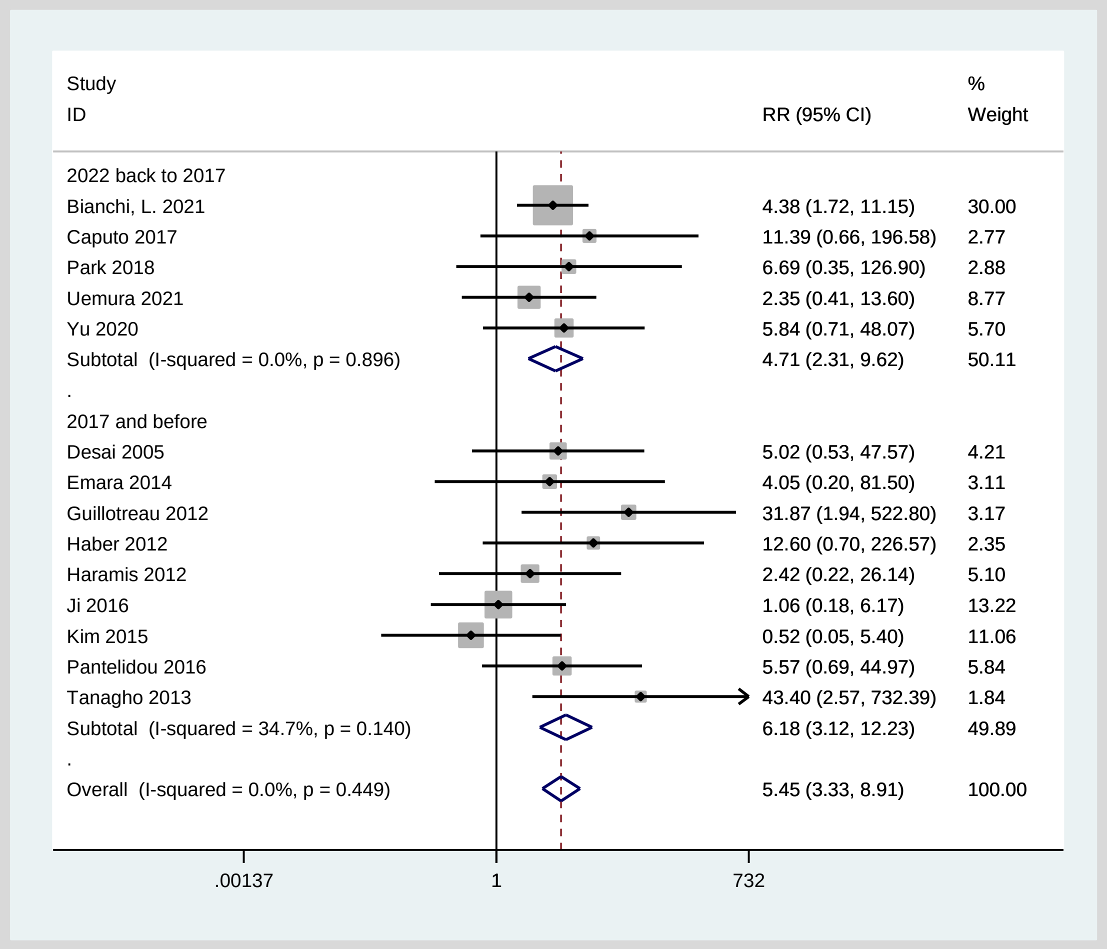

c) Given that LPN has been largely supplanted by Robotic Partial nephrectomy (https://pubmed.ncbi.nlm.nih.gov/19616229/, https://www.karger.com/Article/Fulltext/508519, https://www.liebertpub.com/doi/10.1089/end.2020.0151) I suggest for separate analysis of LPN vs focal therapy, and RPN vs focal therapy.

We subgroup analysis by LPN vs focal therapy, and RPN vs focal therapy. These studies found no differences.

Operating time (OP):

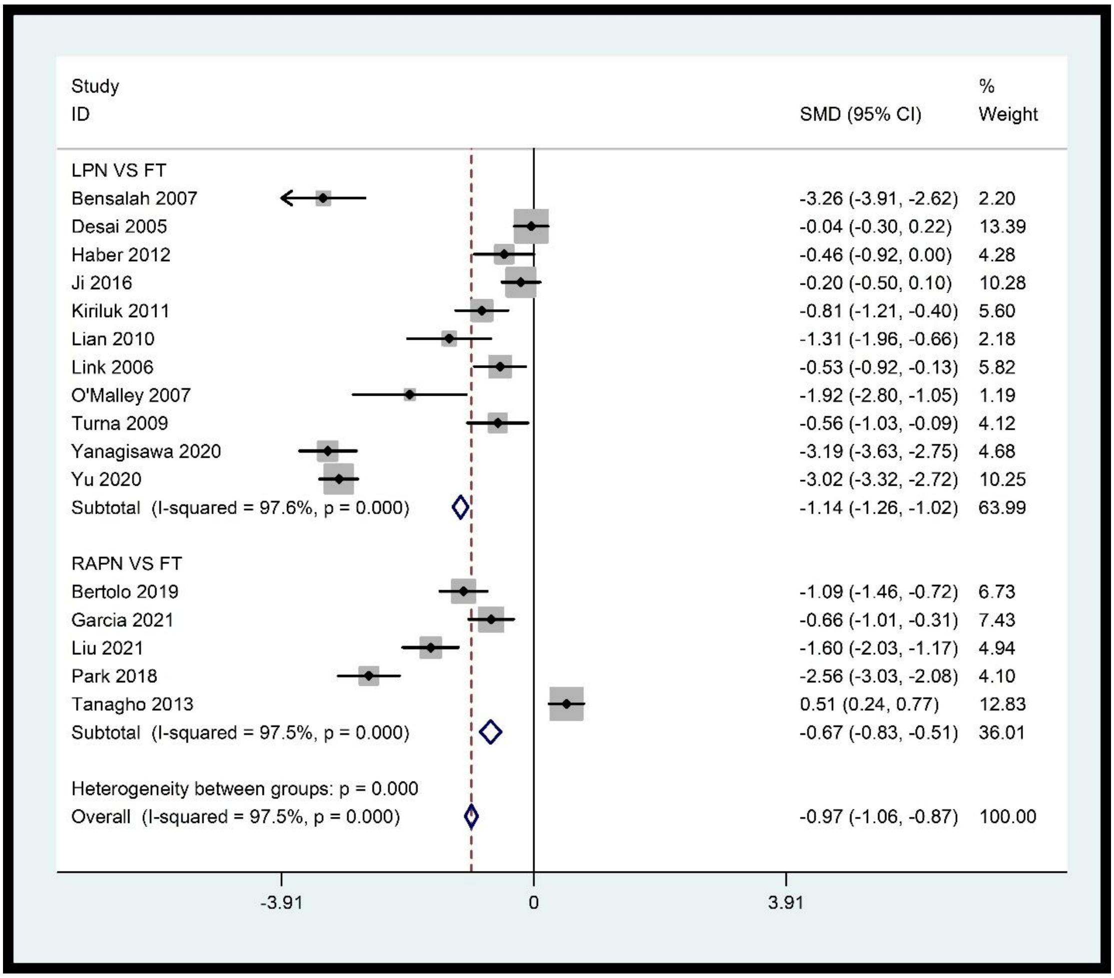

Estimated blood loss (EBL):

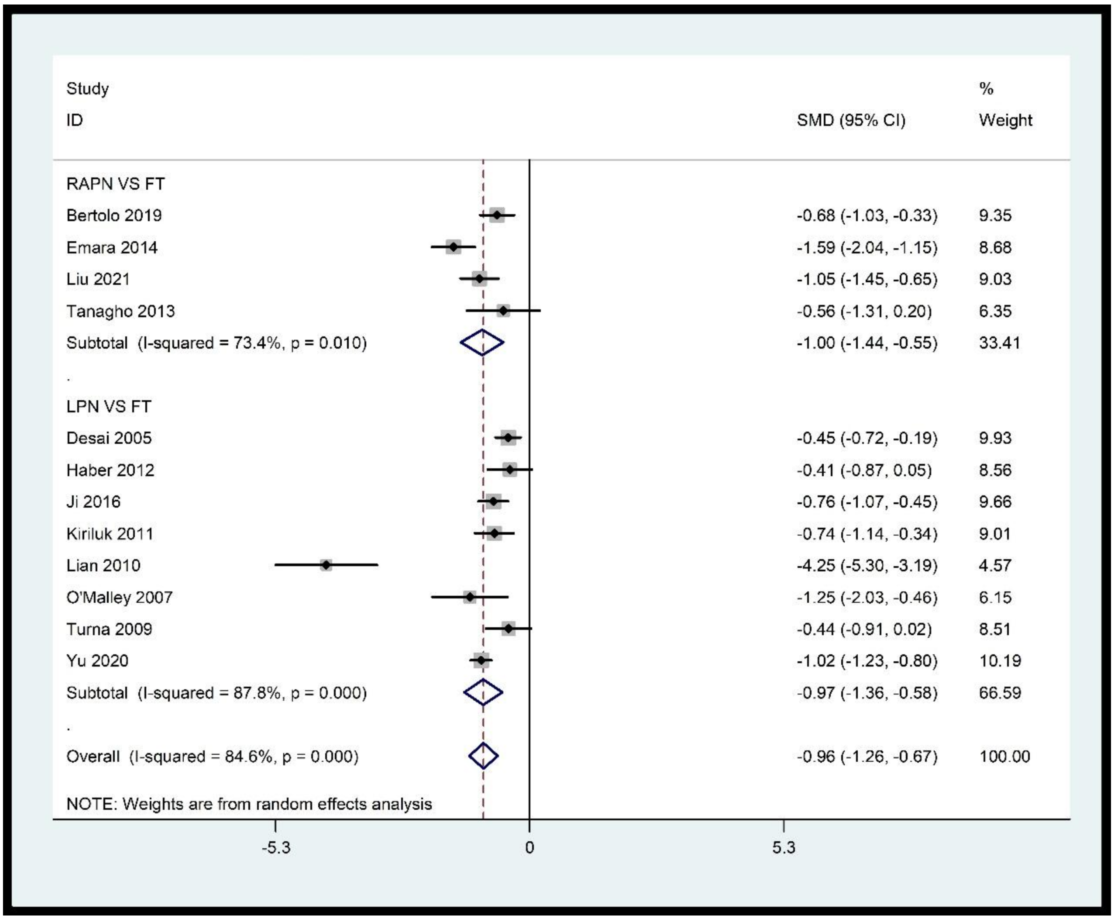

Length of stay (LOS):

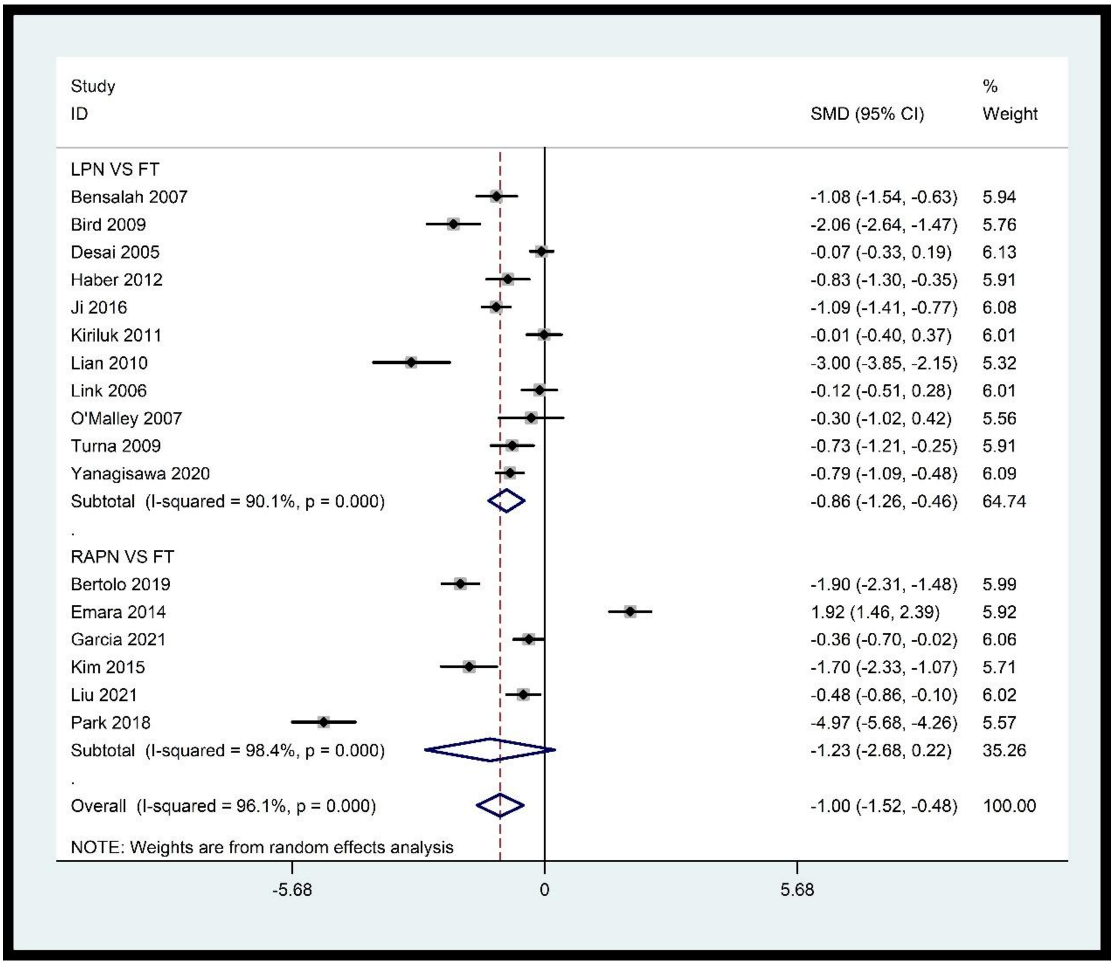
Study
ID
SMD (95% CI)
%
Weight
LPN VS FT
Bensalah 2007 -1.08 (-1.54, -0.63) 5.94
Bird 2009 -2.06 (-2.64, -1.47) 5.76
Desai 2005 -0.07 (-0.33, 0.19) 6.13
Haber 2012 -0.83 (-1.30, -0.35) 5.91
Ji 2016 -1.09 (-1.41, -0.77) 6.08
Kiriluk 2011 -0.01 (-0.40, 0.37) 6.01
Lian 2010 -3.00 (-3.85, -2.15) 5.32
Link 2006 -0.12 (-0.51, 0.28) 6.01
O'Malley 2007 -0.30 (-1.02, 0.42) 5.56
Turna 2009 -0.73 (-1.21, -0.25) 5.91
Yanagisawa 2020 -0.79 (-1.09, -0.48) 6.09
Subtotal (I-squared = 90.1%, p = 0.000) -0.86 (-1.26, -0.46) 64.74
.
RAPN VS FT
Bertolo 2019 -1.90 (-2.31, -1.48) 5.99
Emara 2014 1.92 (1.46, 2.39) 5.92
Garcia 2021 -0.36 (-0.70, -0.02) 6.06
Kim 2015 -1.70 (-2.33, -1.07) 5.71
Liu 2021 -0.48 (-0.86, -0.10) 6.02
Park 2018 -4.97 (-5.68, -4.26) 5.57
Subtotal (I-squared = 98.4%, p = 0.000) -1.23 (-2.68, 0.22) 35.26
.
Overall (I-squared = 96.1%, p = 0.000) -1.00 (-1.52, -0.48) 100.00
NOTE: Weights are from random effects analysis
-5.68
0
5.68

GFR:

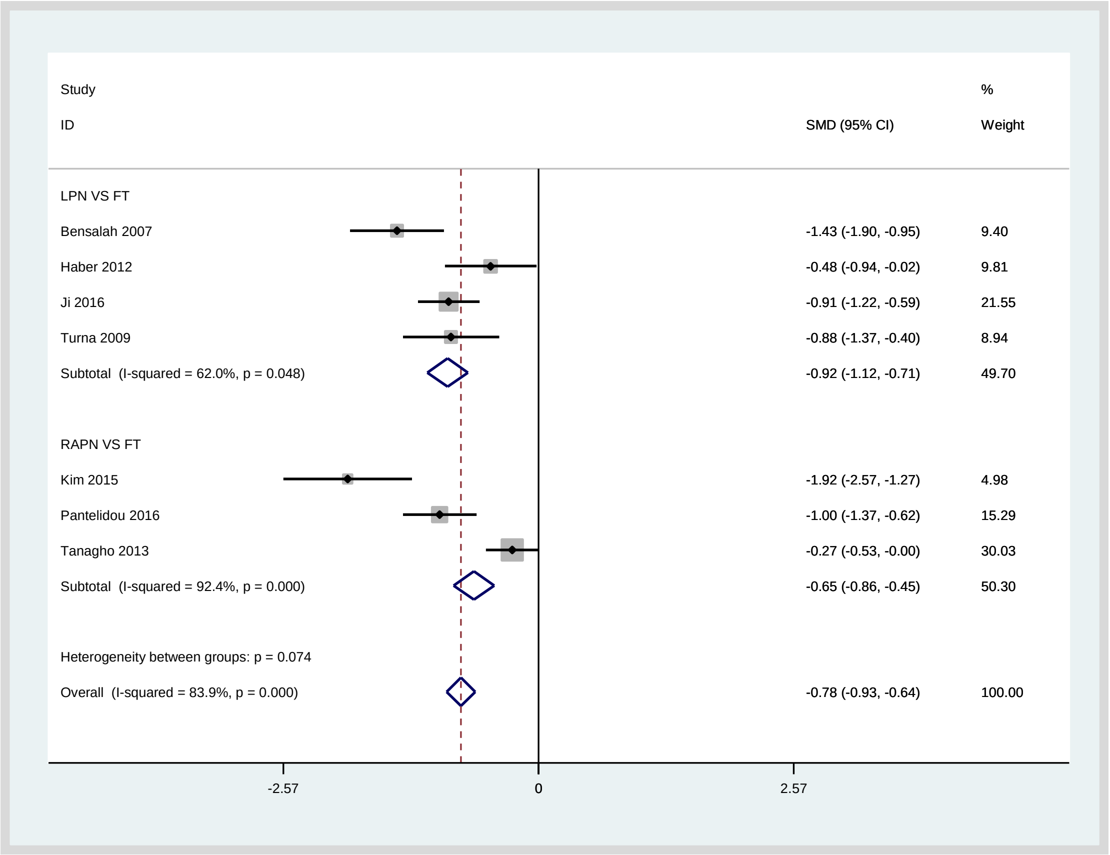

Distant metastasis:

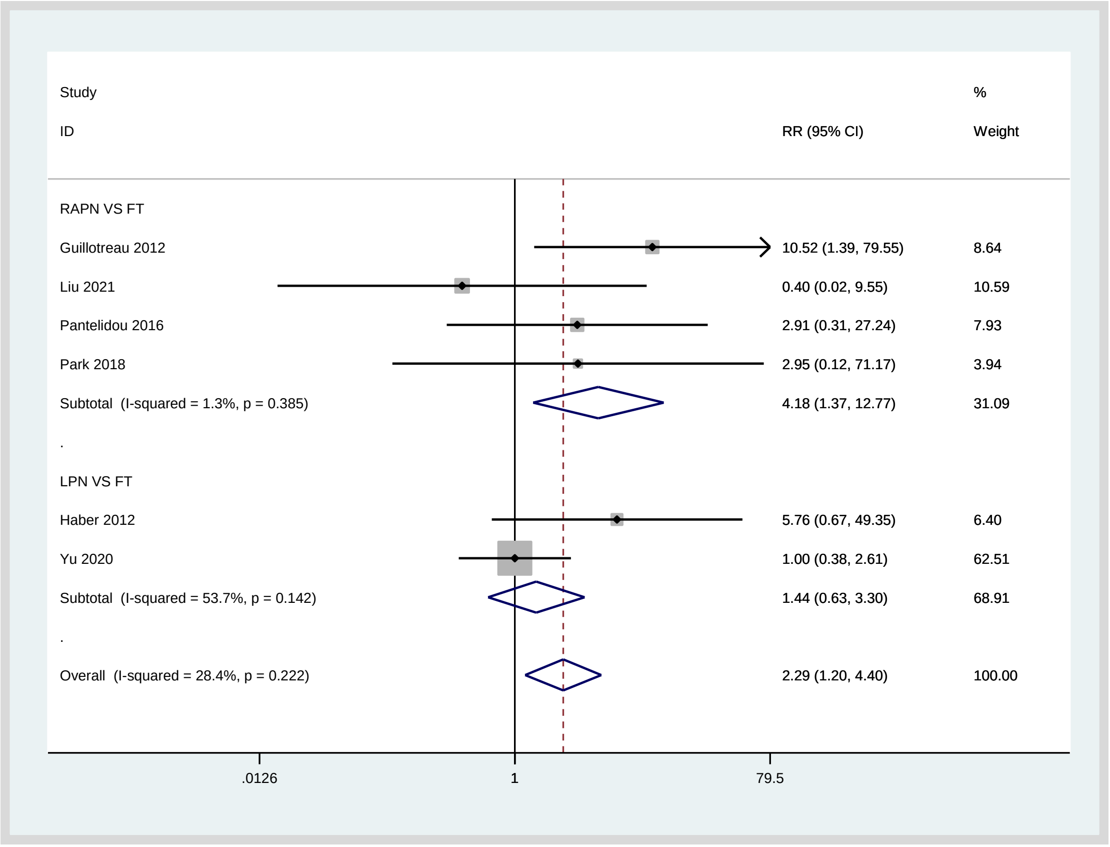

Local recurrence:

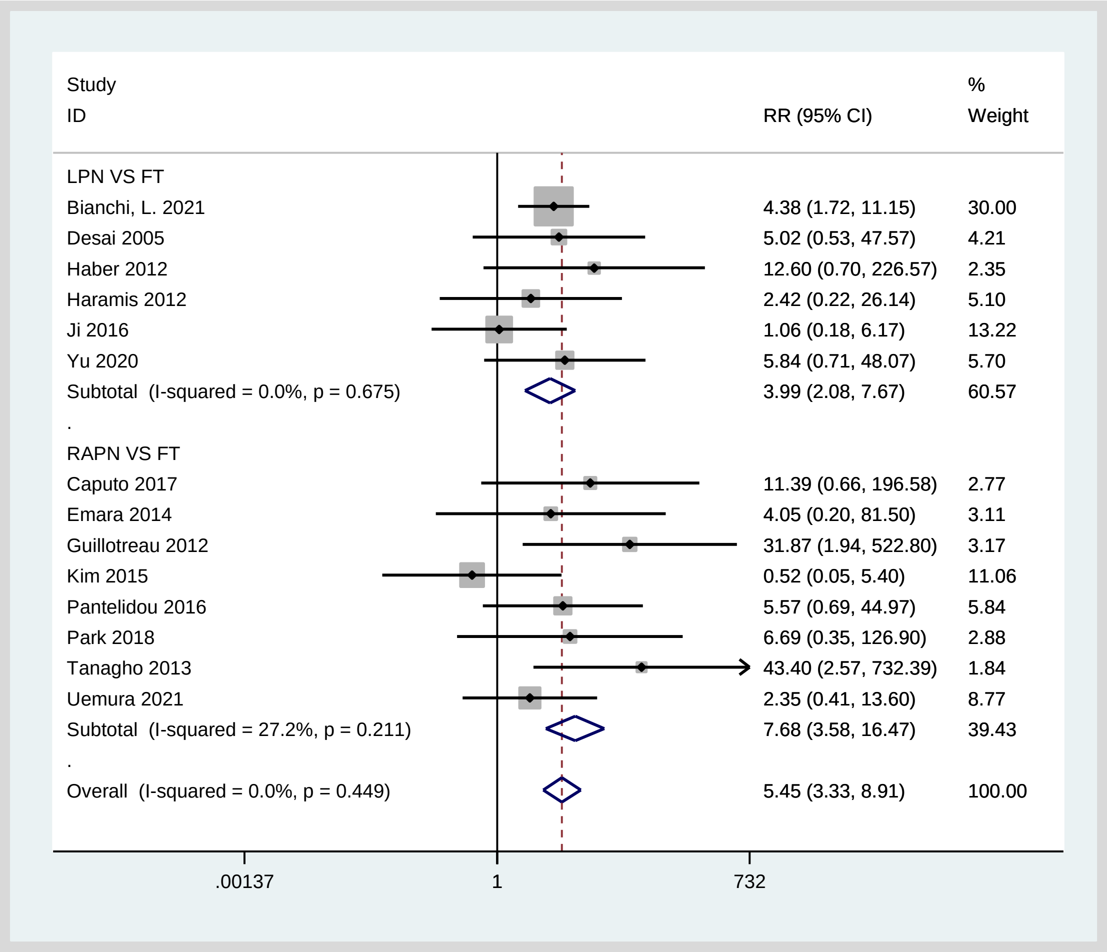

Supplement: Supplementary file 2 [file DataSheet_1_v1.pdf]
